# A preS2 aa1–26-specific humoural response marks functional cure in chronic HBV infection

**DOI:** 10.1016/j.ebiom.2026.106355

**Published:** 2026-06-29

**Authors:** You-Yuan Wang, Jun-Liang Fu, Wen-Xin Wang, Hong-Min Wang, Shan-Quan Liu, Ying Sun, Zi-Wei Wang, Jing Li, Ming-Ju Zhou, Wei-Zhe Li, Meng-Meng Zhu, Xia Li, Yu-Xuan Yang, Si-Yuan Chen, Tao Yang, Xing Fan, Ruo-Nan Xu, Yan-Mei Jiao, Jin-Wen Song, Cheng Zhen, Min Zhang, Ming Shi, Fu-Sheng Wang, Chao Zhang

**Affiliations:** a Senior Department of Infectious Diseases, Chinese PLA General Hospital, Beijing, China; b PLA Medical School, Beijing, China

**Keywords:** HBV infection, Functional cure, Humoural immunity, preS2, PhIP-seq

## Abstract

**Background:**

Functional cure (FC) of chronic hepatitis B (CHB) is closely associated with restoration of HBV-specific humoural immunity, yet the epitope-resolved features of humoural immunity recovery during interferon-induced FC remain unknown.

**Methods:**

We profiled HBV-specific humoural responses in 65 patients with CHB drawn from three independent PEG-IFNα-treated cohorts, including nucleos(t)ide analogue-treated patients with viral suppression and inactive HBsAg carriers. Linear epitope mapping was performed using phage immunoprecipitation sequencing (PhIP-seq), with key findings validated by longitudinal ELISA and complemented by *ex vivo* phenotypic characterisation of epitope-specific B cells using fluorescent peptide tetramers.

**Findings:**

PhIP-seq identified 297 reactive peptides, with a marked enrichment of antibody reactivity toward the HBsAg preS domain in patients who achieved FC. Longitudinal analysis demonstrated that preS2-directed antibodies, particularly those targeting the N-terminal amino acids 1–26 (preS2 aa1–26), increased progressively and closely parallelled HBsAb seroconversion, consistently distinguishing FC from non-FC group. In parallel, *ex vivo* B-cell profiling revealed that preS2 aa1–26-specific B cells exhibited a plasmablast-skewed and IgG-dominant profile, in contrast to the more IgM-biased and less differentiated phenotypes observed in preS1- and SHBs-specific B cells. Within the preS2-specific compartment, FC showed higher CXCR5 and CD69 expression than non-FC group, indicative of enhanced maturation and improved follicular homing potential.

**Interpretation:**

PreS2 aa1–26 may serve as a key humoural HBV-specific epitope associated with functional cure, with potential implications for immunotherapeutic strategies in CHB.

**Funding:**

This work was supported by the National Science and Technology Major Project (2025ZD01905905), National Key Research and Development Program of China (2022YFA1303600 and 2023YFC2308100), and the National Natural Science Foundation of China (82130019 and 82272311).

**Research in context**

**Evidence before this study**

Functional cure of chronic hepatitis B (sustained HBsAg loss) is rarely achieved with nucleos(t)ide analogues but occurs in a subset of patients treated with PEG-IFNα. While functional cure is associated with the recovery of HBV-specific B-cell immunity, previous studies have largely focussed on global antibody levels (total anti-HBs) or broad B-cell defects. The specific linear epitopes targeted during successful seroclearance, and the phenotypic characteristics of B cells recognising these precise epitopes, have not been fully defined.

**Added value of this study**

Using unbiased phage immunoprecipitation sequencing (PhIP-seq), we identified that antibodies specific to the preS2 aa1–26 epitope are consistently associated with functional cure, in contrast to antibodies targeting the major S-loop (aa124–147). We demonstrate that preS2 aa1–26-specific B cells in patients with functional cure exhibit a class-switched (IgG+), plasmablast-like phenotype, which is distinct from the B-cell states observed against other HBsAg regions. Immunofluorescence analysis of liver tissue indicates that preS2-containing HBsAg remains detectable on hepatocyte membranes, including in core-negative contexts consistent with expression from integrated HBV DNA, and may represent an accessible antigenic target for humoural immune recognition.

**Implications of all the available evidence**

These findings refine our understanding of humoural immunity in CHB by showing that epitope specificity and B-cell quality are distinct features of functional cure, separate from total antibody quantity. The identification of the preS2 aa1–26–directed response offers a specific immunological feature that may help to characterise heterogeneity in PEG-IFNα treatment responses. The study highlights the potential relevance of immune responses targeting antigen products derived from integrated HBV DNA in the context of HBsAg clearance.

## Introduction

Chronic hepatitis B (CHB) affects over 296 million people worldwide and causes approximately 820,000 annual deaths due to liver cirrhosis and hepatocellular carcinoma (HCC).^1^ Functional cure (FC) of CHB, defined as sustained loss of hepatitis B surface antigen (HBsAg) after a finite course of therapy with or without seroconversion to HBsAb, remains the optimal treatment endpoint to prevent disease progression.^2^ Among the existing treatment modalities, pegylated interferon alpha (PEG-IFNα) remains a key agent for achieving a functional cure. The combination of PEG-IFNα with investigational novel agents has been shown to result in elevated levels of HBsAg clearance.^3^, ^4^, ^5^ Further elucidating the immunological characteristics of PEG-IFNα therapy is crucial for optimising current functional cure strategies and improving treatment outcomes.^6^^,^^7^

HBV-specific humoural immunity plays an indispensable role in the control of HBV infection.^8^^,^^9^ The presence of HBsAb reliably indicates recovery from acute HBV infection and effective vaccination.^10^ In patients with CHB, baseline HBsAb-secreting B cells can predict HBsAg or HBeAg seroconversion during treatment,^11^ and higher post-treatment HBsAb titres are associated with durable FC.^12^ However, during chronic HBV infection, HBsAg-specific B cells frequently exhibit functional exhaustion, characterised by reduced proliferative capacity and impaired differentiation into antibody-secreting plasma cells.^13^^,^^14^ Although several studies suggest that humoural function can be partially restored during FC,^15^, ^16^, ^17^, ^18^ the epitope-level features of this recovery, particularly which B-cell targets are preferentially reactivated, remain unclear.

HBsAg is composed of three co-terminal envelope proteins; depending on the initiating site, they are categorised as small (SHBs), medium (preS2 and SHBs, MHBs) or large (preS1, preS2 and SHBs, LHBs) surface proteins.^19^ Neutralising antibodies classically target the SHBs “α-determinant”, blocking viral attachment to hepatocyte heparan sulphate proteoglycans, or conserved motifs in preS1 that inhibit sodium taurocholate co-transporting polypeptide (NTCP) mediated viral entry.^20^^,^^21^ However, current investigations of humoural responses in FC have focussed predominantly on SHBs.^22^^,^^23^ In contrast, high-resolution, longitudinal epitope mapping of preS1/preS2, particularly when paired with matched B-cell phenotyping during HBsAg loss, remains lacking, leaving the contribution of preS-directed immunity to PEG-IFNα-mediated humoural recovery poorly defined.

Phage immunoprecipitation sequencing (PhIP-seq) epitope identification coupled with epitope-specific B-cell tetramer flow cytometry enables high-resolution mapping of linear antibody epitopes together with direct identification and phenotyping of cognate B cells.^24^ Applying this integrated approach to longitudinal samples from PEG-IFNα-treated patients can therefore reveal epitope-level humoural signatures that track with functional cure. In this study, using PhIP-seq discovery, ELISA validation and tetramer-based B-cell profiling, we delineate epitope-level humoural features that underpin PEG-IFNα-induced HBsAg seroclearance. Our findings may serve both as a biomarker for patient stratification and as a candidate target for antibody-based therapeutics.

## Methods

### Study participants

Cohort 1 included patients with CHB who had received nucleos(t)ide analogue (NA) treatment for ≥1 year, achieved serum HBsAg <1500 IU/mL and HBV DNA <20 IU/mL. Patients were excluded if they had a coinfection with human immunodeficiency virus, hepatitis A virus, hepatitis C virus, hepatitis D virus, or hepatitis E virus; the presence of other chronic liver disease or serious systemic diseases; IFN allergy; and receiving IFN, glucocorticoids, or other immunomodulatory therapy within 6 months prior to enrolment. 32 patients were enrolled (Table S1). Participants were treated with either entecavir (ETV) or tenofovir disoproxil fumarate (TDF) in combination with PEG-IFNα (180 μg weekly) for 48 weeks. After a 48-week post-treatment follow-up, 18 patients met FC criteria and 14 did not. Peripheral blood samples were collected at weeks 0, 24, 48 and 60.

Cohort 2 comprised inactive HBsAg carriers (IHC), defined as untreated individuals with chronic HBV infection who had persistently normal ALT, HBsAg <1500 IU/mL, and HBV DNA <2000 IU/mL. The exclusion criteria matched those of Cohort 1. Nineteen participants were enrolled (Table S2). Participants were administered a combination of tenofovir alafenamide fumarate (TAF) and PEG-IFNα (180 μg weekly) for 48 weeks. After a 48-week follow-up, 12 were classified as FC and 7 as non-FC. Peripheral blood sampling followed the same schedule as Cohort 1.

Cohort 3 was a cross-sectional cohort consisting of 14 patients treated with NA meeting the same eligibility criteria as Cohort 1. Based on the 48-week follow-up after PEG-IFNα cessation, 7 participants were classified as FC and 7 as non-FC. Specifically, 12 participants were sampled at the end of PEG-IFNα therapy, 1 participant was sampled 12 weeks before the end of therapy and 1 participant was sampled 12 weeks after the end of therapy. Clinical characteristics are summarised in Table S3.

Liver biopsies were obtained from 9 treatment-naïve HBeAg-positive patients. Clinical characteristics are summarised in Table S4.

This study enrolled 3 participants who achieved FC without PEG-IFNα and 5 healthy controls (HC). Among HCs, 2 participants received a single dose of a second-generation recombinant hepatitis B vaccine (20 μg) before sampling. The remaining 3 participants had not received any hepatitis B vaccine within the preceding five years. Clinical characteristics are summarised in Table S5.

The study cohort consists of an ethnically homogenous Chinese population and sex was self-reported by participants at enrolment. All participants in this study were enrolled from the Fifth Medical Center of Chinese PLA General Hospital (Beijing, China).

### Design and cloning of HBV peptide library sequences

The HBV protein sequence information was searched and downloaded from the Uniprot database, and non-redundant protein sequences were obtained after removing duplicate entries (including different fragments of the same protein). Each protein was divided into a number of peptide segments with a length of 56 aa and 50% overlap of adjacent peptide segments along the direction from the N-terminal to the C-terminal; peptides at the end of the C-terminal segment that were less than 56 aa were extended to 56 aa toward its N-terminal; and protein sequences that were less than 56 aa in length were complemented at their N-terminal with the GSGS … sequence. The final library encompassed 5301 non-redundant HBV peptides covering the entire HBV proteome (Table S6). The peptide sequences were converted into DNA sequences with codon optimisation, giving priority to codons with high frequency in *Escherichia coli* and avoiding enzymatic sites used in vector construction, and further optimised with a GC content in the range of 40–60%. After that, a common sequence of 16 bp was added at both ends of the DNA sequence for subsequent PCR amplification and vector construction. The synthesised oligo DNA library was dissolved and quantified and 2 ng of the template was quantitatively extracted. Primers were designed and synthesised based on the consensus sequence for oligo library amplification, followed by gel extraction. The recovered product was subjected to double digestion and ligated with a phage vector at 16 °C for 16 h. Subsequently, the packaging system was added, and the reaction was carried out at 22 °C for 2 h. The reaction was terminated by adding 90 μL of antibiotic-free medium. Products that passed the packaging efficiency assay were subjected to large-scale amplification. The supernatant was collected by centrifugation for titre determination.

### Phage immunoprecipitation and sequencing

Plasma samples were centrifuged at 12,000 rpm for 20 min at 4 °C. The supernatant was collected and diluted 1:1000 with PBST buffer to a final volume of 1 mL, then transferred to pre-blocked 96-deep-well plates (each plate included negative control wells, blank control wells, and positive control wells). Diluted phage libraries were added to each well and placed on a rotator for overnight incubation at 4 °C. After incubation, 20 μL of Protein G magnetic Beads (Smart lifescience, SM036050) were added for 4 h on a rotator at 4 °C. Following incubation, the plate was centrifuged at 500 g for 3 min and then placed on a 96-well magnetic stand to immobilise the Protein G magnetic beads, discard the solution, and add 200 μL TBS (containing 0.1% IPEGAL CA 630, Sigma, I3021) to each well to resuspend the beads; this washing step was repeated twice. The beads were resuspended in 40 μL of sterile water and transferred to a new 96-well PCR plate. After sealing the plate, it was immediately centrifuged for 10 s and heated at 95 °C for 10 min in a PCR thermocycler. Sequencing library preparation was performed using a two-round PCR amplification strategy. The first round employed universal primers matching phage sequences, while the second round utilised primers containing barcodes and adaptor sequences. PCR products from 1 to 3 96-well plates were pooled (3 μL per well), and target bands were isolated via gel electrophoresis followed by gel extraction. High-throughput sequencing was subsequently conducted using an Illumina sequencing platform and corresponding reagent kits. Each sample was sequenced to a depth of 4 million reads, with a read length of 150 bp. Full-length sequences were obtained by merging paired-end reads, and the read count per peptide served as the primary quantitative metric.

### Analysis of PhIP-seq data

The sequencing data were analysed with a slight modification of a previous study.^25^, ^26^, ^27^ Briefly, the sequencing volume of different samples was normalised (the sequencing volume of each sample was adjusted to 1.25 M reads) to obtain the Normalised reads (NR) of each peptide in each sample, based on the initial library sequencing abundance, the number of input reads for each peptide, the signal Enrichment factor (EF) of different peptides in each sample was further calculated, finally, the P-value of each peptide was calculated using a generalised Poisson distribution model. The thresholds for determining positively reactive HBV peptides were set to NR > 15, EF > 1.5 and −log10 (P-value) > 1 (Figure S1). The number of positive-reactive peptides (peptide hits) in HBV antigen was calculated to compare whether there were differences between the FC and non-FC groups. The PhIP-seq sequencing was conducted by Beijing Boao Jingdian Biotechnology Co., Ltd.

### ELISA for detecting HBV antigen-specific antibodies

Recombinant HBV antigen preS1 (P7401), preS2 (P7406) and SHBs (P7392) were purchased from Beyotime, and preS2-related peptides (BSA conjugation) were synthesised by MCE. HBV antigen or individual peptides (10 μg/mL) were coated in the high-binding flat-bottom polystyrene microplate (Corning, 9018) at 4 °C overnight. After washing 3 times with PBST buffer (MCE, HY-K1025), the plate was blocked by rapid blocking buffer (MCE, HY-K1027) to avoid non-specific binding, and plasma samples (1:100 diluted with PBS) were added to the plates, incubated at 4 °C for about 12 h. The plate was then washed 5 times with PBST buffer and incubated with horseradish peroxidase (HRP)-conjugated goat anti-human IgG-Fc antibody (300 ng/mL in PBS, ABCAM, ab97225) for 1 h at room temperature, then washed the plate 5 times again with PBST buffer. Add 100 μL of TMB (Beyotime, P0208) sequentially and incubate at room temperature for about 10 min. After stopping the reaction by the stop solution for the TMB substrate (Beyotime, P0215), the optical density (OD) value was measured at 450 nm using a microplate reader (Synergy HTX, Bio-Tek).

### Multiplex immunofluorescence

Multiple immunofluorescences (mIF) staining of liver tissues with reference to previous studies.^28^ Briefly, formalin-fixed, paraffin-embedded (FFPE) liver biopsy slides were baked for 2 h at 60 °C before staining. Slides were rehydrated with a series of graded ethanol solutions in deionised water. Antigen retrieval was performed at pH 6 for 20 min at 95 °C. Slides were serially stained with the following antibodies: anti-Core (ZSGB-Bio, OTI1E8), -SHBs (ZSGB-Bio, OTI1D3), -preS2 (Arigo, SQab1507). Anti-mouse horseradish peroxidase (Cell Signalling Technology, #7076) was used as the secondary antibody with an incubation time of 10 min. TSA-conjugated fluorophores (PerkinElmer, NEL741) were used to visualise each biomarker, consisting of 480 (HBcAg), 570 (SHBs) and 620 (preS2), with incubation times for each primary antibody being 1 h. Slides were mounted with anti-fade mounting medium (Life Technologies, P36931) and stored at 4 °C before imaging. Images of whole slides were captured (40x magnification for multispectral images) using the phenoimager HT 2.0 multispectral imaging platform (Akoya Biosciences), and the images were analysed using the inForm Tissue Analysis Software (Akoya Biosciences), with cell percentages calculated and locations labelled.

### Construction of HBV-peptide tetramers

HBV peptide tetramers were constructed based on previous studies.^29^^,^^30^ Briefly, the target peptide was synthesised with biotinylated modification on the C-terminal, and the biotinylated HBV antigen was incubated with PE-labelled streptavidin (Agilent, PJRS25-1) at room temperature for at least 1 h. Then, remove unbound antigen from the solution using a 100 kDa Ultra-0.5 Centrifugal Filter (Millipore, UFC5100). The final concentration was measured using Nanodrop in the UV–Vis setting on the instrument. APC-labelled streptavidin (Agilent, PJ27S-1) not incubated with antigen was used as a decoy to exclude cells with non-specific binding to the tetramer backbone during flow cytometry staining.

### HBV-specific B cell flow cytometry

Peripheral blood was collected in ethylenediaminetetraacetic acid-anticoagulated tubes. Peripheral blood mononuclear cells (PBMCs) were isolated using Ficoll-Hypaque density-gradient centrifugation. Isolated PBMCs were stored in liquid nitrogen. Before performing flow cytometry, PBMCs were resuspended and incubated in RPMI 1640 containing 10% foetal bovine serum (FBS) at 37 °C and 5% CO2 for 2 h. Subsequently, the cell concentration was adjusted to 1 × 10^6^ cells/mL, followed by staining with Fixable Viability Stain 700 (1:1000 dilution, BD Biosciences) for 15 min in sodium azide- and protein-free Dulbecco's Phosphate Buffered Saline (1X DPBS) at room temperature. HBV-specific B cell surface markers and functional molecules stained with monoclonal antibodies (mAbs) and HBV peptides tetramers (1 μM/mL) for 30 min at 4 °C. Staining antibodies were listed in Table S7. The stained samples were examined by flow cytometry on a BD A5 flow cytometer (BD Biosciences). Flow cytometry data were analysed using FlowJo software (version 10.10.0, BD Biosciences).

### Statistics

Statistical analyses and image processing were performed using R (version 4.4.1). Data were shown as median with interquartile ranges or mean with 95% confidence interval. The difference between the two groups was decided by using the t-test for normally distributed data or the Mann–Whitney U test for non-normally distributed data. Similarly, for correlation analysis, Pearson's r test was used if the data followed a normal distribution; otherwise, Spearman's r test was used. Differences are considered significant at ∗p < 0.05, ∗∗p < 0.01, ∗∗∗p < 0.001, and ∗∗∗∗p < 0.0001 levels.

### Ethics statement

This study involves human participants and was approved by the Ethics Committee of the Fifth Military Medical Centre of the Chinese PLA General Hospital (approval number KY-2024-4-58-1). Written informed consent was obtained from all participants in accordance with the Declaration of Helsinki before inclusion.

### Role of funders

The funders had no role in the design of the study, data collection, analysis or preparation of the manuscript.

## Results

### Comprehensive profiling of HBV-specific antibody reactivity using PhIP-seq

**Fig. 1:**
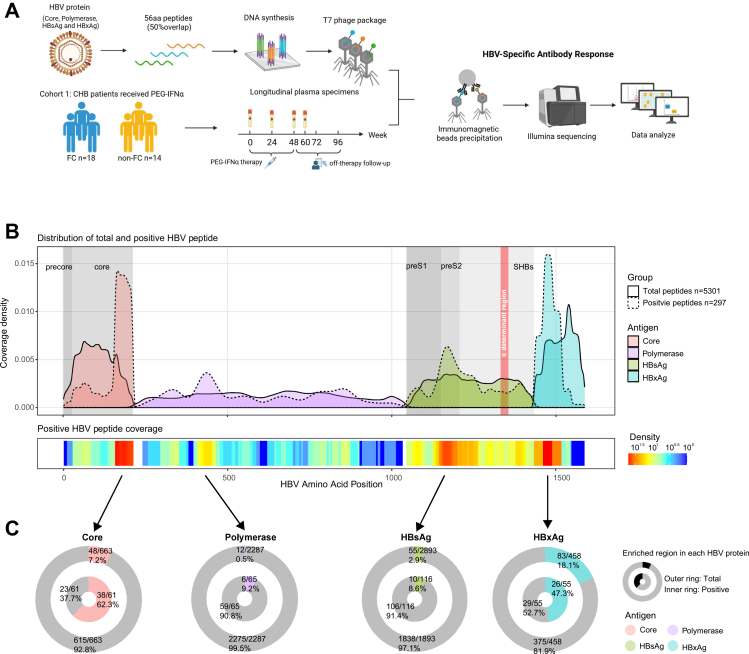
**Overall characteristics of HBV PhIP-seq.** (A) Schematic representation of the HBV PhIP-seq workflow. Sampling time points included week 0 (baseline prior to PEG-IFNα treatment), week 24 (mid-treatment), week 48 (end of treatment), and week 60 (12 weeks post-treatment). (B) Density plot (top) and heatmap (bottom) showing the distribution characteristics of reactive peptides (n = 297, dashed line) compared with the full HBV peptide library (n = 5301, solid line). (C) The proportion of positive peptide aggregation regions within each antigen (inner ring) compared with their proportional representation in the HBV peptide library (outer ring). The aggregation regions for Core, Polymerase, HBsAg and HBxAg are shown from left to right. FC, functional cure.

To systematically delineate antibody response patterns during PEG-IFNα therapy, we performed PhIP-seq profiling in 32 patients with CHB (Cohort 1), generating 117 longitudinal plasma samples collected at baseline (week 0), on-treatment (week 24), end of treatment (week 48), and off-treatment follow-up (week 60) (Fig. 1A, Figure S1A–E, Table S1). Among the 5301 HBV-derived peptides represented in the screening library, 297 showed positive reactivity (Figure S1F). Although the PhIP-seq library provides near-uniform coverage across the HBV genome, the reactive peptide set displayed a clearly skewed distribution. At the antigen level, reactivity was markedly enriched in Core and HBxAg but under-represented in Polymerase, while the proportion mapping to HBsAg remained comparable to the library background (Figure S1G). Moreover, reactive peptides clustered into distinct immunoreactive hotspots within individual antigens (Fig. 1B). Specifically, preferentially targeted regions included amino acids (aa) 157–212 of Core (62.3% vs 7.2%), aa 197–252 of Polymerase (9.2% vs 0.5%), aa 102–157 of HBsAg (8.6% vs 2.9%), and aa 29–84 of HBxAg (47.3% vs 18.1%) (Fig. 1C). Notably, the enriched Core region precisely aligned with previously defined immunodominant B-cell epitopes,^23^ supporting the technical reliability of PhIP-seq. Together, these data demonstrate that PhIP-seq enables high-resolution identification of HBV immunogenic hotspots, revealing distinct regional enrichment patterns across the viral proteome.

### Dynamic patterns of antibody reactivity during PEG-IFNα therapy in FC and non-FC patients

**Fig. 2:**
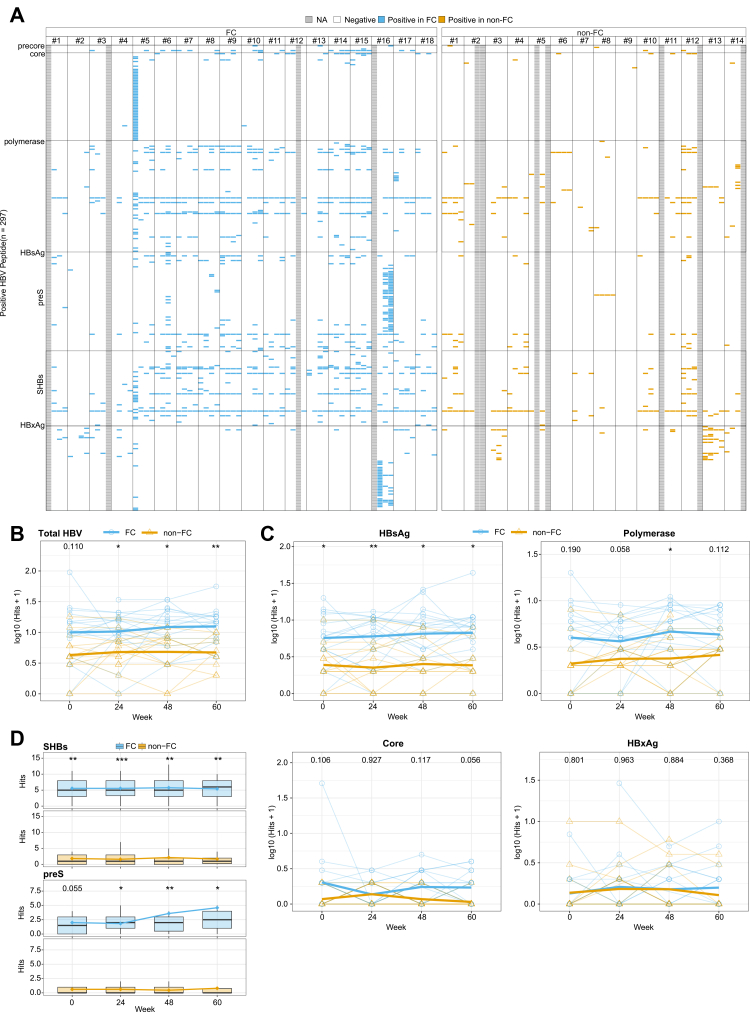
**Comparison of HBV antibody responses between FC and non-FC groups by HBV peptidehits.** (A) Heatmap displaying the distribution of 297 positively reactive peptides across 117 longitudinal samples from 32 participants. (B) Intergroup comparisons of total HBV peptide hits at four time points. Thin lines represent individual trajectories; thick lines indicate group means. (C) Intergroup comparison of peptide hits stratified by antigen (HBsAg, Core, Polymerase, HBxAg). Thin lines represent individual trajectories; thick lines indicate group means. (D) Dynamic patterns of SHBs peptide hits (top) and preS peptide hits (preS1 + preS2, bottom) in FC and non-FC groups. Statistical analysis was performed using the t-test and the p values were adjusted by the Bonferroni method (B and C), and the Mann–Whitney U test (D). ∗p < 0.05; ∗∗p < 0.01; and ∗∗∗p < 0.001. FC, functional cure.

After establishing the overall landscape, we next compared longitudinal antibody reactivity in patients who achieved functional cure (FC) versus those who did not (non-FC). A heatmap of peptide reactivity demonstrated notable inter-individual heterogeneity, with the FC group generally exhibiting a higher count of reactive peptides (Fig. 2A). Over the course of therapy, the number of peptide hits progressively increased in the FC group but remained relatively stable in non-FC patients (Fig. 2B). When stratified by antigen category, the HBsAg-specific antibody response was consistently higher in FC patients at all time points, whereas no marked group differences were observed for other antigens, except for a transient polymerase-specific separation at week 48 (Fig. 2C). These results indicate that the antibody advantage associated with the FC group is predominantly concentrated within the HBsAg region.

Given the distinct biological roles of preS (preS1 and preS2) and SHBs domain,^31^, ^32^, ^33^ we further analysed these components separately (Fig. 2D). The mean value of antibody reactivity against preS increased in the FC group but showed minimal change in the non-FC group, with significant intergroup differences emerging at weeks 24, 48, and 60. By contrast, SHBs-specific responses differed between groups at baseline and subsequently followed nearly parallel trajectories in both groups, resulting in a persistent intergroup gap over the course of therapy (Fig. 2D). Together, these results imply that the antibody response against preS differs from SHBs, demonstrating an increasing trend during PEG-IFNα treatment in patients undergoing HBsAg clearance.

### Associations between antibody responses and HBsAg/HBsAb levels

**Fig. 3:**
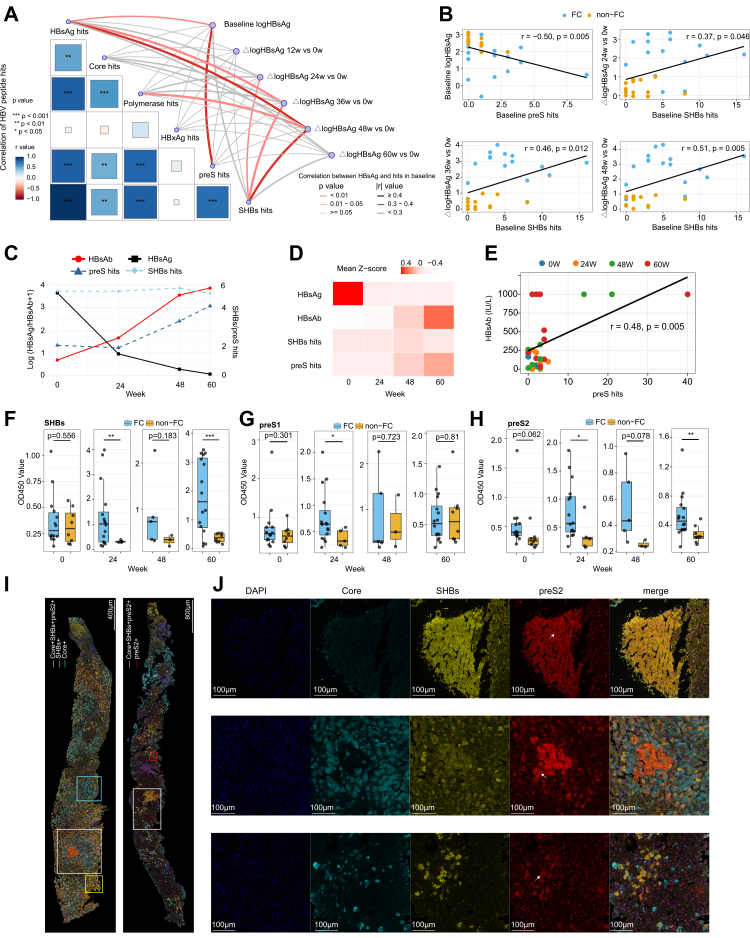
**Anti-preS2 is associated with HBsAb in the FC group.** (A) Pairwise correlations among antigen-specific peptide hits (bottom left) and correlations with baseline clinical variables (top right). (B) Correlation of baseline preS and SHBs peptide hits with baseline HBsAg levels. (C) Longitudinal trajectories of HBsAg, HBsAb, preS hits, and SHBs hits in the FC group. (D) Heatmap showing Z-scored mean values of HBsAg, HBsAb, SHBs hits, and preS hits across four time points. (E) Correlation between quantitative HBsAb and preS peptide hits in the FC group. (F–H) ELISA OD450 values for SHBs, preS1, and preS2 antibodies at weeks 0, 24, 48, and 60. The number of samples available for testing varied across time points, week 0: FC (n = 14) and non-FC group (n = 8); week 24: FC (n = 16) and non-FC group (n = 5); week 48: FC (n = 5) and non-FC group (n = 3); and week 60: FC (n = 16) and non-FC group (n = 8). SHBs (F), preS1 (G), and preS2 (H). (I) Representative images of mIF showing overlaid Core, SHBs and preS2 antigen staining distribution pattern of liver tissues. Left, scale bar = 400 μm; Right, scale bar = 800 μm. (J) Representative images of differential cell localisation of Core, SHBs and preS2 antigen expression. The white arrows marked preS2 expression in the liver cell membrane. Core (cyan), SHBs (yellow), preS2 (red) and DAPI (blue). Scale bar = 100 μm. Statistical analysis was performed using Spearman's r test (A, B, E) and t-test (F–H). ∗p < 0.05; ∗∗p < 0.01; ∗∗∗p < 0.001. FC, functional cure; mIF, multiplex immunofluorescence.

To further assess the clinical relevance of antibody reactivities, we next examined the correlations between antigen-specific peptide hits and HBsAg and HBsAb levels. At baseline, antibody responses to preS, SHBs, and other HBV antigens (except HBxAg) showed strong internal co-correlations (Fig. 3A). Notably, baseline antibody response against preS was inversely correlated with baseline HBsAg levels (r = −0.50, p = 0.005, Spearman's r test), suggesting that stronger preS-directed responses may reflect a more favourable pre-treatment immune-viral setpoint. In contrast, baseline antibody response against SHBs did not correlate with baseline HBsAg, but instead showed positive correlations with the magnitude of on-treatment HBsAg decline (24w, r = 0.37, p = 0.046; 36w, r = 0.46, p = 0.012; 60w, r = 0.51, p = 0.005, Spearman's r test) (Fig. 3A and B), indicating a potential link with antigen clearance dynamics.

We then focussed on the FC group to evaluate how preS- and SHBs-specific responses related to longitudinal HBsAg and HBsAb trajectories. In FC group, the increase in preS peptide hits closely parallelled rising HBsAb levels (Fig. 3C and D), and preS hits were positively correlated with quantitative HBsAb (r = 0.48, p = 0.005, Spearman's r test) (Fig. 3E). These observations suggest that the preS region may contain epitopes associated with the activation of humoural immunity, which may be associated with successful antigen clearance.

To validate the PhIP-seq findings, we performed ELISA-based quantification of antibodies targeting SHBs, preS1, and preS2. Consistent with the sequencing data, antibody responses to SHBs and preS2 were significantly higher in FC compared with non-FC patients across multiple time points (Fig. 3F–H). At baseline, among the three tested antigens, only the antibody response to preS2 showed a significant inverse correlation with HBsAg levels (r = −0.478, p = 0.03, Spearman's r test) (Figure S2A–C). Correlation analyses at week 60 further demonstrated positive associations between HBsAb levels and SHBs- and preS2-specific responses, with preS2 showing the highest correlation (preS2, r = 0.849, p < 0.001; SHBs, r = 0.543, p = 0.084, Spearman's r test) (Figure S2D–F). Anti-preS2 levels were also evaluated in HCs (as negative control) and participants who achieved FC without PEG-IFNα treatment (Figure S2G). Although anti-preS2 responses in these individuals were lower than those observed in the FC group of Cohort 1, detectable reactivity remained present more than five years after HBsAg seroclearance and remained above the levels observed in HCs. Collectively, these findings identify preS2-directed antibody responses as the most robust serological correlate of both HBsAg decline and HBsAb development during functional cure of CHB.

We also examined the distribution of HBV antigens in liver tissue to clarify whether preS2-specific antibodies could exert their effects intrahepatically. The three antigens—Core, SHBs, and preS2—were largely co-distributed within HBV-infected hepatocytes, with a notable overlap between preS2 and SHBs expression (Figure S2H). Nevertheless, each antigen could also be expressed individually in hepatocytes (Fig. 3I). Importantly, preS2 antigen was detectable on the cell membrane, enabling specific antibodies to bind to hepatocytes expressing preS2 (Fig. 3J). Furthermore, among preS2-positive cells, both HBV-replicating hepatocytes (Core+ SHBs+) and HBV-integrated hepatocytes (Core− SHBs+) were present (Figure S2H). In summary, based on the expression and distribution characteristics of preS2 antigen in the liver, preS2-specific antibodies can also bind to antigens expressed on the hepatocyte membrane and thereby exert their effects.

### Identification of humoural immune epitopes within the preS2 region

**Fig. 4:**
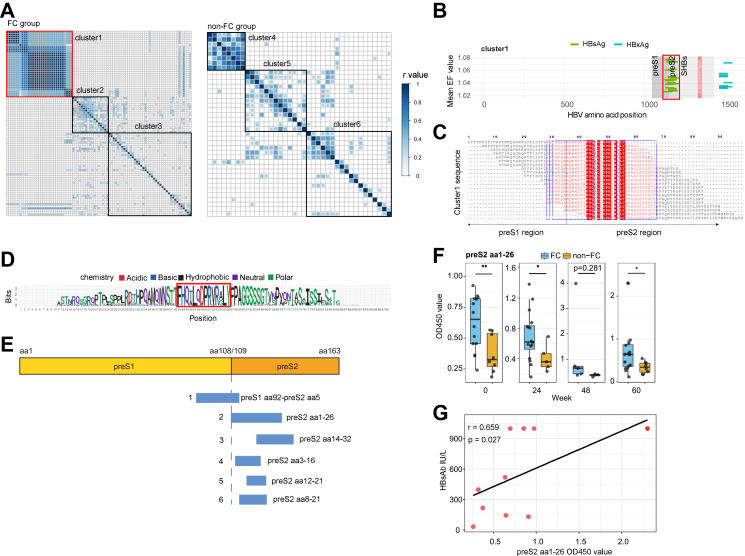
**The N-terminal of the preS2 region contained humoural immune epitopes associated with the HBsAb seroconversion.** (A) Correlation-based clustering of positively reactive peptides in FC (left) and non-FC (right) groups. The colour of each square denoted the strength of the correlation between peptides, and each black box represented a cluster generated by hcluster. (B) Mapping of Cluster 1 peptides to HBV antigens in the FC group. (C) Sequence alignment of the 21 peptides within cluster 1. (D) Full-length sequence logo of the 21 peptides within cluster 1, the red frame indicates the fully overlapping region. (E) Sequence and positional characteristics of six synthetic peptides selected for ELISA validation. (F) ELISA OD450 values for anti-preS2 aa1–26 antibodies across four time points. (G) Correlation between anti-preS2 aa1–26 responses and HBsAb levels at week 60. Statistical analysis was performed using t-test (F) and Spearman's r test (G). ∗p < 0.05; ∗∗p < 0.01. EF, enrichment factor; FC, functional cure.

Given the pronounced intergroup differences in preS2-directed responses, we next sought to map humoural epitopes within this region. Hierarchical clustering revealed six distinct clusters (Fig. 4A). Notably, the FC group showed both a greater number of reactive peptides and stronger intra-cluster correlations, with Cluster 1 emerging as a uniquely enriched immunoreactive signature in this group (Fig. 4A). Examining the peptide positional distribution (Fig. 4B and Figure S3A) showed that peptides in Cluster 1 from FC group were localised within the preS2-associated segment of HBsAg and HBxAg. Specifically, the preS2-associated region contained 21 reactive peptides spanning a contiguous 98-aa stretch (Fig. 4C and D, Table S8). Within this segment, a fully overlapping 14-aa motif was shared across peptides, suggesting a highly conserved humoural hotspot (Fig. 4D). Consistent with findings in Fig. 3E, reactivity intensity for Cluster 1 peptides exhibited a positive association with HBsAb levels (Figure S3B). Furthermore, both the response frequency and degree for these 21 peptides were substantially higher in FC than in non-FC group (Figure S3C–E). Together, these findings indicate that the N-terminal portion of preS2 represents a key humoural target linked to HBsAg seroconversion in individuals with FC.

Since the N-terminal region of preS2 has been identified as a dominant antibody binding site,^34^, ^35^, ^36^ we refined the epitope resolution by aligning the 21 Cluster 1 peptides to the UniProt reference and synthesising targeted peptides based on the representative HBV genotype B sequence (P17398) (Table S9). Incorporating previously reported epitopes together with our PhIP-seq findings,^34^^,^^37^, ^38^, ^39^, ^40^ we selected several candidate peptides across the preS2 domain for ELISA validation (Fig. 4E and Table S10). ELISA results demonstrated that the antibody responses targeting preS2 aa1–26 region showed statistically significant intergroup differences at three follow-up time points. It positively correlated with HBsAb at week 60 (r = 0.659, p = 0.027, Spearman's r test) and exhibited a negative correlation trend with HBsAg at week 0 (Fig. 4F and G, Figure S4A). Within this region, peptides aa12–21 and aa8–21—both embedded within aa1–26—also showed significant intergroup differences at two time points, whereas other candidate epitopes displayed only transient or minimal discrimination (Figure S4B–F). Similar to the pattern observed for anti-preS2 (Figure S2G), anti-preS2 aa1–26 responses remained lower in individuals who achieved FC without PEG-IFNα treatment than in the FC group of Cohort 1, but higher than those in negative controls (Figure S4G). Collectively, PhIP-seq and ELISA results converge to identify preS2 aa1–26 as the most prominent humoural epitope associated with functional cure in CHB.

### Independent validation of antibody responses targeting preS2 aa1–26

**Fig. 5:**
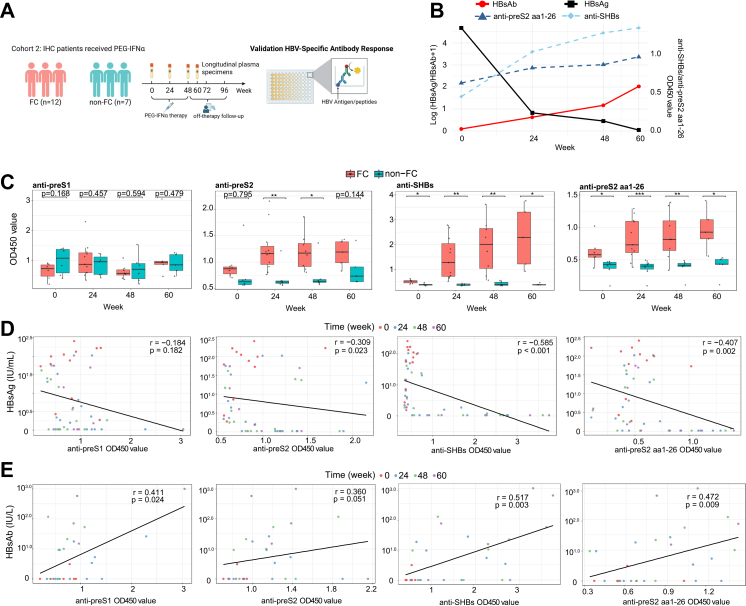
**Validation of preS2 aa1–26 humoural immunogenicity in inactive HBsAg carriers.** (A) Schematic of ELISA testing in Cohort 2. (B) Longitudinal trajectories of HBsAg, HBsAb, anti-SHBs, and anti-preS2 aa1–26 levels. (C) Intergroup comparison of antibody responses to preS1, preS2, SHBs, and preS2 aa1–26. The number of samples available for testing varied at different time points, week 0: FC (n = 6) and non-FC group (n = 6); week 24: FC (n = 11) and non-FC group (n = 7); week 48: FC (n = 8) and non-FC group (n = 6); and week 60: FC (n = 5) and non-FC group (n = 5). (D) Correlation analysis between the levels of HBsAg and antibody responses targeting preS1, preS2, SHBs, and preS2 aa1–26. (E) Correlation analysis between HBsAb levels in FC group and antibody responses targeting preS1, preS2, SHBs, and preS2 aa1–26. Statistical analysis was performed using t-test (C) and Spearman's r test (D and E). ∗p < 0.05; ∗∗p < 0.01; ∗∗∗p < 0.001. FC, functional cure.

To assess the robustness and generalisability of our findings, we validated preS2 aa1–26-specific antibody responses in an independent longitudinal cohort consisting of 54 plasma samples from 19 inactive HBsAg carriers (IHC) treated with PEG-IFNα (Cohort 2, 12 FC and 7 non-FC, Fig. 5A, Table S2, Figure S5A–E). In FC group, longitudinal analysis revealed that anti-preS2 aa1–26 closely tracked with rising HBsAb levels, mirroring the relationship observed in Cohort 1 (Fig. 5B and Figure S5F). Comparative analysis of antibody responses against preS1, preS2, SHBs, and the preS2 aa1–26 epitope revealed distinct patterns. Anti-preS2 responses showed significant intergroup differences at weeks 24 and 48, whereas anti-SHBs responses differed between groups at all four assessed time points (Fig. 5C). Notably, anti-preS2 aa1–26 responses discriminated FC from non-FC at every time point, underscoring the robustness of this epitope-level signal (Fig. 5C). Correlation analyses further supported these observations. Anti-preS2, anti-SHBs, and anti-preS2 aa1–26 levels were inversely correlated with quantitative HBsAg, while anti-preS1, anti-SHBs, and anti-preS2 aa1–26 levels positively correlated with HBsAb in patients undergoing HBsAg clearance (Fig. 5D and E). Furthermore, to ascertain the generalisability of antibody responses targeting amino acids 1–26 of preS2 in predicting HBsAg seroconversion, we combined antibody response data from Cohort 1 and Cohort 2 at baseline of PEG-IFNα therapy. The predictive efficacy of the preS2 1–26 amino acid epitope antibody response (AUC = 0.857) significantly outperformed that of the three HBsAg antigens, demonstrating the epitope's substantial potential in predicting HBsAg seroconversion (Figure S5G). Collectively, these results demonstrate that the preS2 aa1–26-directed antibody response is consistently enriched in patients who achieve functional cure and that its longitudinal trajectory closely parallels HBsAb development across distinct pre-treatment phases of HBV infection.

### B-cell tetramers enable the identification of HBV epitope-specific B cells *ex vivo*

**Fig. 6:**
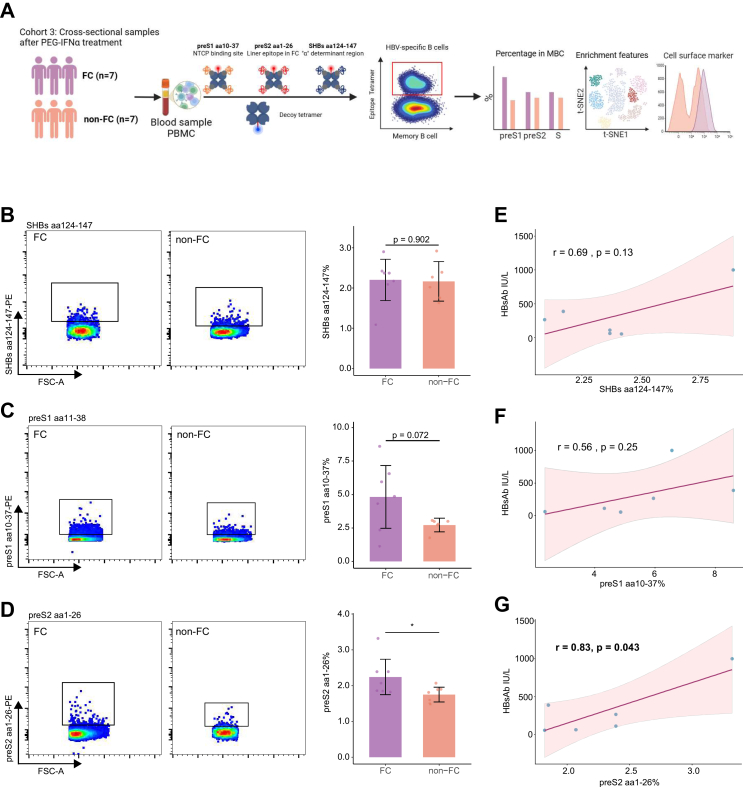
**Difference in frequency of the HBsAg epitope-specific B cells.** (A) The overall flowchart of HBsAg epitope-specific B cell flow cytometric analysis from PEG-IFNα-treated patients (n = 14). (B–D) Representative flow plots (left) and group comparisons (right) of preS2 aa1–26-specific (B), SHBs aa124–147-specific (C), and preS1 aa10–37-specific (D) B cells. Data are presented as mean ± 95% CI. (E–G) Correlation analysis between epitope-specific B-cell frequencies and HBsAb levels in FC patients. preS2 aa1–26-specific B cells (E), SHBs aa124–147-specific B cells (F), and preS1 aa10–37-specific B cells (G). Statistical analysis was performed using t-test (B–D) and Pearson's r test (E–G). ∗p < 0.05. FC, functional cure. MBC, memory B cells. NTCP, sodium taurocholate co-transporting polypeptide.

Elucidating HBV epitopes associated with functional cure provides an opportunity to dissect key features of protective humoural immunity. To directly interrogate epitope-specific B cells, we analysed 14 patients who received PEG-IFNα-treatment in Cohort 3, including 7 who achieved HBsAg clearance (FC) and 7 non-FC individuals (Fig. 6A and Table S3). Consistent with Cohorts 1 and 2, FC group in Cohort 3 also mounted stronger antibody responses against the preS2 aa1–26 region (Figure S6A and B). Guided by the well-established peptide-based tetramer approaches for the identification and phenotypic characterisation of antigen-specific B cells, which have been widely applied across viral infection and vaccination settings^24^^,^^29^^,^^30^ and informed by both our PhIP-seq–defined epitope landscape and prior knowledge of HBV surface antigen biology,^31^^,^^41^ we generated fluorescent B-cell tetramers targeting three biologically and functionally relevant linear epitopes within HBsAg: preS2 aa1–26 (the dominant epitope defined by PhIP-seq), preS1 aa10–37 (neutralising epitope in NTCP-binding domain in genotype B), and SHBs aa124–147 (the classical α-determinant neutralising region), HBsAb-negative HCs were used as negative controls for flow cytometry staining (Fig. 6A and Figure S7A and B). Across the three tetramers, only preS2 aa1–26-specific B cells showed a clear FC versus non-FC difference, whereas preS1-specific B cells displayed only a modest elevation in the FC group and SHBs-specific B cells showed no separation between groups (Fig. 6B–D). Importantly, frequencies of preS2 aa1–26-specific B cells were strongly associated with quantitative HBsAb levels in FC patients (r = 0.83, p = 0.043, Pearson's r test), while preS1- and SHBs-specific B cells demonstrated no such relationship (Fig. 6E–G). Furthermore, the frequency of preS2 aa1–26-specific B cells demonstrated substantial concordance with antibody response in both FC and non-FC groups, reflecting the reliability of the specific B-cell detection methodology (Figure S7C). Together, these results mirror the antibody-level observations and establish preS2 aa1–26 as the dominant B-cell epitope associated with PEG-IFNα-induced functional cure.

### Distinct features of epitope-specific B cells in FC versus non-FC patients

**Fig. 7:**
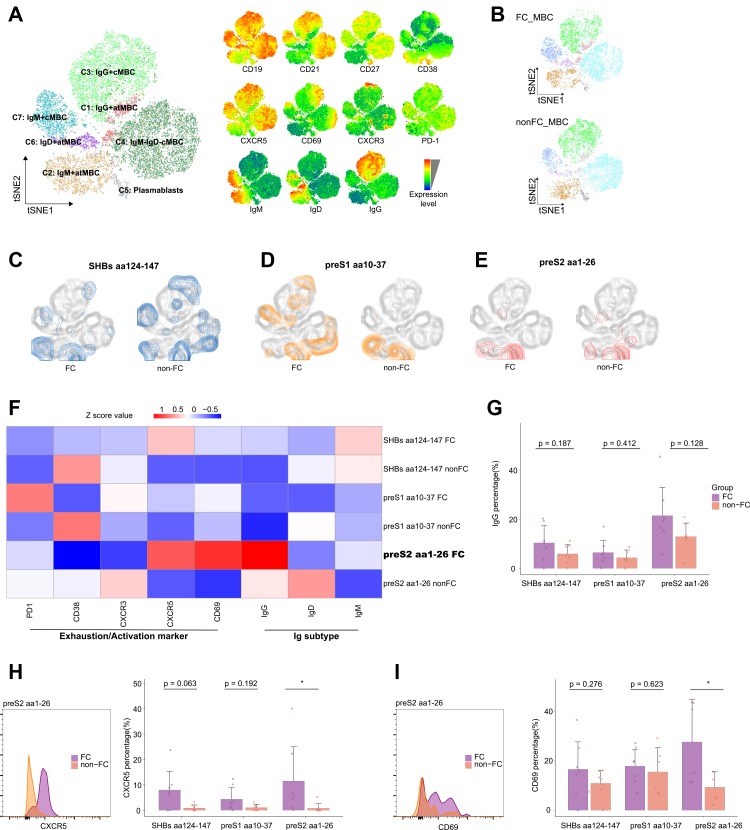
**Distribution and surface marker characteristics of HBsAg-specific B cells.** (A) t-SNE projection of concatenated memory B cells from 14 PEG-IFNα-treated patients, with 7 MBC subsets delineated by expression of 11 markers. (B) The t-SNE plot of MBC in the FC group (top) and the non-FC group (bottom). (C–E) Overlay of epitope-specific B cells onto the global MBC landscape for SHBs aa124–147 (C), preS1 aa10–37 (D), and preS2 aa1–26 (E). (F) Z-score values of relative expression levels of eight surface markers in three epitope-specific B cell subsets between the FC and non-FC Groups. The colour intensity in the heatmap represents only the inter-group differences within each cell surface marker and does not reflect the expression levels across different markers. (G) The proportions of IgG among the 3 HBsAg-specific B cells in the FC and non-FC groups. (H–I) Group comparisons of CXCR5 (H) and CD69 (I) expression in preS2 aa1–26-specific B cells, with representative flow cytometry profiles shown on the right. Data are presented as mean + 95% CI and statistical analysis was performed using t-test (G–I). ∗p < 0.05. FC, functional cure. MBC, memory B cells.

To resolve the phenotypic diversity of HBV epitope-specific B cells, we applied t-SNE to 24,000 concatenated memory B cells from FC and non-FC groups in Cohort 3. Based on 11 surface markers, 7 canonical subsets were identified, including three clusters of classical memory B cells (cMBCs, CD21 high, CD27 high), three clusters of atypical memory B cells (atMBCs, CD21 low, CD27 low), and a plasmablast cluster (CD27 high, CD38 high) (Fig. 7A and B and Figure S7D–F). Antigen-specific subset analysis revealed a striking divergence between epitopes. PreS1 aa10–37-specific and SHBs aa124–147-specific B cells from non-FC patients were predominantly enriched within IgM^+^ atypical memory B cells, a hallmark population of chronic viral infection characterised by diminished BCR signalling and reduced effector potential (Fig. 7C and D, Figure S7F). In contrast, preS2 aa1–26-specific B cells from both FC and non-FC groups were highly concentrated within the plasmablast compartment, with 68.8% of preS2-specific B cells in FC group displaying plasmablast features, indicating active differentiation into antibody-secreting cells (Fig. 7E and Figure S7F).

We next examined activation and differentiation marker expression across epitopes. In an intra-comparison of different cell surface markers, all three epitope-specific B cell types from non-FC group expressed higher levels of CD38, consistent with chronic activation, metabolic stress, and impaired effector maturation (Fig. 7F, Figure S7G). Phenotypically, preS2 aa1–26-specific B cells in FC group displayed a more mature and activated profile—including higher IgG expression, increased CXCR5, and elevated CD69—whereas their counterparts in non-FC group exhibited a muted activation phenotype (Fig. 7G–I). Together with the antibody data, these findings highlight a coordinated preS2-specific B cell differentiation and antibody response program as a central component underlying functional cure in chronic HBV infection.

## Discussion

Humoural immunity is a central component of antiviral defence in chronic HBV infection. In PEG-IFNα-treated CHB, pre-existing HBV-specific antibodies and B-cell responses have been associated with more favourable treatment outcomes.^11^ Concurrently, PEG-IFNα therapy has been demonstrated to restore both the quantity and functionality of HBsAg-specific humoural immunity.^15^ However, the specific viral epitopes that define successful humoural immunity—and their relationship to functional cure—have remained poorly characterised. By integrating high-resolution PhIP-seq mapping, ELISA validation, and *ex vivo* epitope-specific B-cell profiling across three clinical cohorts, this study identifies preS2 aa1–26 as a major humoural epitope linked to HBsAg seroclearance and delineates its underlying B-cell phenotypic features.

Using PhIP-seq across longitudinal samples, we mapped 297 reactive HBV peptides spanning four HBV antigens and observed that major humoural differences between FC and non-FC groups were concentrated within HBsAg. Among the HBsAg domains, antibodies targeting preS2, particularly the N-terminal preS2 aa1–26 segment, showed the strongest association with treatment outcomes. Stronger preS2 responses were associated with lower baseline HBsAg and higher post-treatment HBsAb levels, suggesting that preS2-directed immunity may reflect a more favourable immune–viral equilibrium before therapy and a more competent humoural response during PEG-IFNα-induced clearance. Importantly, the preS2 aa1–26 signal was independently reproduced in a second clinical cohort, underscoring its robustness and generalisability as a dominant correlate of functional cure.

Previous studies comparing the antibody responses between FC and non-FC and consistently reported higher antibody titres among those achieving HBsAg loss.^16^^,^^22^ However, earlier work primarily focussed on the SHBs region of HBsAg, especially the classical α-determinant, where dominant neutralising epitopes reside. While these epitopes undoubtedly contribute to viral control, accumulating serological evidence indicates that multiple sequences within preS2 can also elicit antibody responses in chronic HBV infection.^38^^,^^39^^,^^42^ Our findings expand the current understanding of humoural immunity in CHB by identifying preS2-directed responses as the humoural feature most closely associated with PEG-IFNα-induced HBsAg seroclearance. In particular, preS2 aa1–26 emerged as a previously underrecognised FC-associated epitope that may help identify patients with favourable responsiveness to PEG-IFNα.

Recent work has established that the phenotype and function of HBV-specific B cells are key determinants of humoural immunity in chronic HBV infection.^8^^,^^43^ Yet, most prior studies examined total HBsAg-specific B cells without resolving epitope-level specificity. Leveraging advances in fluorescence-labelled antigen-specific B cell technologies, we used epitope-defined tetramers to compare B-cell responses targeting three biologically relevant HBsAg regions: preS1 aa10–37, preS2 aa1–26, and SHBs aa124–147. This approach enabled a direct dissection of how epitope specificity shapes B-cell differentiation and functional potential.

Across epitopes, our findings reinforce and extend prior observations of profound dysfunction within HBsAg-specific B cells.^8^^,^^14^^,^^43^, ^44^, ^45^ Both preS1-specific and SHBs-specific B cells were largely confined to IgM^+^ atypical memory compartments and expressed elevated PD-1, features indicative of chronic antigenic stimulation and impaired plasmablast differentiation. These patterns suggest that, despite detectable binding, tolerance and exhaustion remain major barriers to effective antibody production in these compartments. In contrast, preS2 aa1–26-specific B cells exhibited two distinguishing features. First, across epitopes, they showed a fundamentally different biology, with strong enrichment in the plasmablast compartment and increased IgG class-switching, features not observed for preS1- or SHBs-specific B cells, suggesting that this subset is comparatively less constrained by the exhaustion program that dominates the broader HBsAg-specific pool.^15^^,^^46^ Second, within the preS2 compartment, patients who achieved FC displayed a more activated and mature phenotype, marked by higher CXCR5 and CD69 expression, whereas non-FC group retained a less differentiated profile. These activation markers have been linked to improved follicular homing and enhanced interactions with follicular helper T cells,^47^ suggesting a more effectively reawakened humoural state during PEG-IFNα therapy. Together, these findings identify preS2-specific B cells as a uniquely responsive humoural axis through which PEG-IFNα overcomes tolerance, with their reactivation closely associated with functional cure.

As the preS2 domain lacks a well-defined hepatocyte receptor for HBV infection, the broader biological relevance of anti-preS2 responses remains incompletely understood.^48^ Nonetheless, the preS2-specific monoclonal antibodies derived from HBV seroconverters predominantly recognise linear epitopes in the N-terminal of the preS2 region are capable of mediating viral neutralisation.^49^ Furthermore, multiple lines of evidence point to its clinical significance. Deletions within preS2 are frequently observed in chronic HBV infection^50^ and are known to reduce viral immunogenicity.^36^ These mutations also correlate with systemic immune exhaustion and are associated with an increased risk of cirrhosis and hepatocellular carcinoma.^51^^,^^52^ Thus, the presence of anti-preS2 antibodies may mark a more preserved antiviral humoural competence.

Although direct mechanistic evidence remains to be established, multiple observations converge on a biologically plausible model that may explain why anti-preS2 responses align more closely with functional cure. In HBeAg-negative CHB, accumulating evidence indicates that the majority of circulating HBsAg is derived from integrated HBV DNA rather than cccDNA transcription,^53^^,^^54^ and PEG-IFNα-based therapy can reduce the level of HBsAg from integrated HBV DNA.^55^ Importantly, integrated sequences predominantly express MHBs and SHBs,^56^^,^^57^ with MHBs constituting a substantial proportion of surface antigen translated from integrated templates.^58^ Notably, MHBs levels decline earlier than SHBs during interferon-induced HBsAg loss.^19^ In parallel, serological studies of acute resolving HBV infection showed that antibodies targeting preS epitopes appear earlier than anti-HBs responses.^59^ Integrating these observations, we propose that PEG-IFNα-mediated immune remodelling may preferentially reduce MHBs, through transiently exposing preS2 epitopes on hepatocytes expressing integrated HBV DNA. Under these conditions, anti-preS2 antibodies may more effectively bind MHBs-positive hepatocytes and engage Fc-dependent effector functions, including antibody-dependent cellular cytotoxicity, antibody-dependent cellular phagocytosis, and enhanced uptake by Fc receptor-positive cells.^60^ These processes may contribute to the clearance of HBsAg-producing hepatocytes during functional cure. These mechanistic inferences will require further experimental validation, particularly regarding how PEG-IFNα modulates MHBs expression dynamics and how anti-preS2 antibodies interface with effector cell subsets *in vivo*.

This study has several limitations. First, PhIP-seq captures only linear epitopes and does not assess conformational antigenicity. Second, phenotypic profiling of epitope-specific B cells was performed cross-sectionally, preventing us from determining whether the observed preS2-specific features reflect baseline differences or interferon-induced remodelling. Third, analyses were restricted to peripheral blood and may not fully reflect intrahepatic humoural immunity. Finally, although the major observations were validated across independent cohorts, some analyses, particularly epitope-specific B-cell profiling, were conducted in relatively small numbers of participants. Additional prospective studies with larger sample sizes and longitudinal sampling will be needed to further establish the immunological and clinical relevance of preS2-directed responses.

In summary, this study identifies preS2 aa1–26 as a key humoural epitope associated with functional cure during antiviral treatment. Epitope-resolved B-cell profiling further reveals that preS2-specific B cells possess a uniquely activated, antibody-secreting phenotype that differentiates FC from non-FC group. Collectively, these findings advance our understanding of HBV-specific humoural immunity in chronic HBV infection and highlight preS2-focussed immunity as a promising target for immune-based therapeutic strategies in CHB.

## Contributors

FSW and CZ (Chao Zhang) conceived the research, supervised the work performed, and worked with YYW and JLF to construct the diagrams and write the manuscript. The participants were enrolled by JLF. The PBMC samples were collected and isolated by WXW, HMW, and SQL. The liver biopsy samples were collected by ZWW and MZ. Experiments were performed by YYW and YS, with technical support of JL, MJZ, WZL, MMZ, XL, and YXY. SYC, TY, XF, RNX, YMJ, JWS, and CZ (Cheng Zhen) edited the manuscript and provided comments and feedback. FSW and MS managed the project team and supervised data collation. FSW and CZ (Chao Zhang) had access to and verified the underlying data. All authors read and approved the final manuscript.

## Data sharing statement

Raw values of PhIP-seq data (including NR, EF and −log10 (P-value)) are available in Supplementary File. The datasets generated during and/or analysed during the current study are available from the corresponding author on reasonable request.

## Declaration of interests

The authors have declared that no conflict of interest exists.

## Appendix A Supplementary data

Supplementary Tables and Figures

−logp raw value

EF raw value

NR raw value

HBV Virus Lib peptides
