# Supplementary material for: A preS2 aa1–26-specific humoural response marks functional cure in chronic HBV infection
Source: eBioMedicine. 2026 Jun 29;129:106355. doi: 10.1016/j.ebiom.2026.106355 (PMC13333318; doi:10.1016/j.ebiom.2026.106355)
Supplement: Supplementary Tables and Figures [file mmc1.docx]

**A preS2 aa1–26-specific humoural response marks functional cure in chronic HBV infection**

Content of supplemental materials

Figure S1………………………………………………………………………….……….2

Figure S2………………………………………………………………………….……….3

Figure S3………………………………………………………………………….……….4

Figure S4………………………………………………………………………….……….5

Figure S5………………………………………………………………………….……….6

Figure S6………………………………………………………………………….……….7

Figure S7………………………………………………………………………….……….8

Table S1……………………………………………………………………………………10

Table S2……………………………………………………………………………………11

Table S3……………………………………………………………………………………12

Table S4……………………………………………………………………………………13

Table S5……………………………………………………………………………………14

Table S6……………………………………………………………………………………15

Table S7……………………………………………………………………………………16

Table S8……………………………………………………………………………………17

Table S9……………………………………………………………………………………18

Table S10…………………………………………………………………………………..19


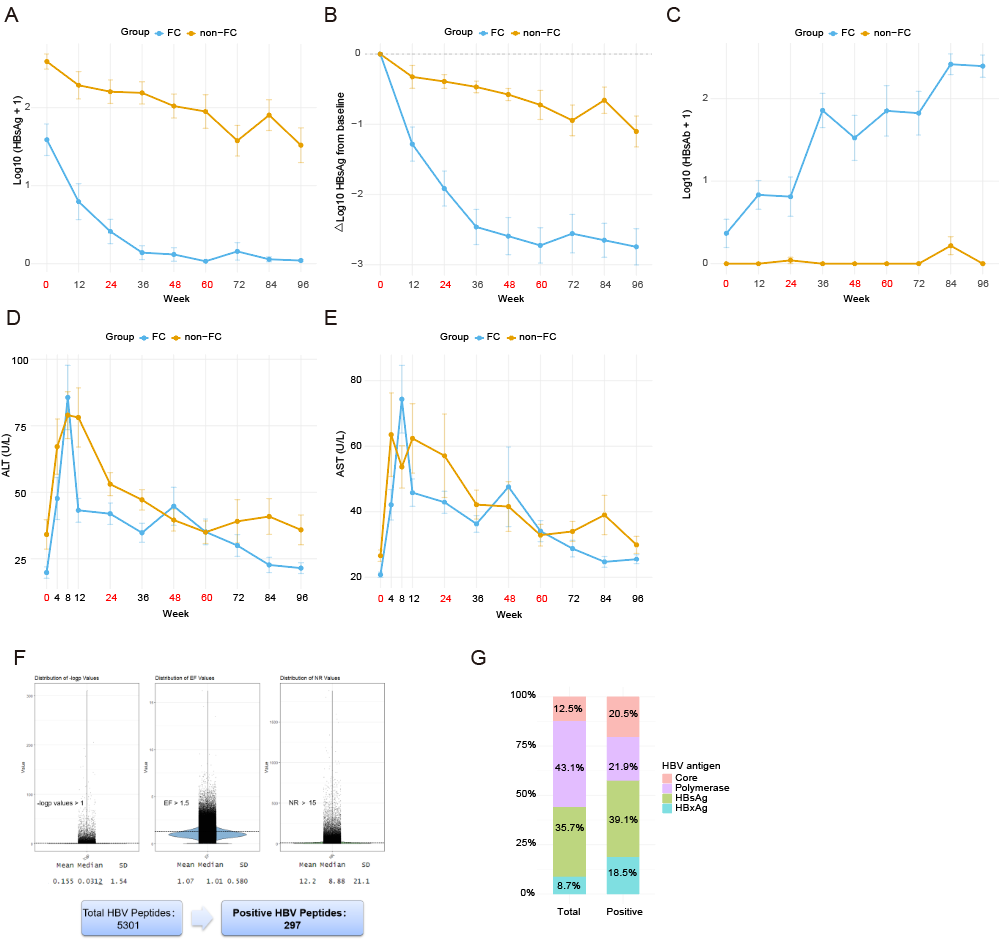


**Figure S1 Screening of plasma samples for positive-reacting peptides from the HBV peptide library.**

(A) Dynamic changes in HBsAg level of Cohort 1 during the follow-up period. (B) The reduction characteristic of the HBsAg level of Cohort 1 during the follow-up period. (C) Dynamic changes of HBsAb level in Cohort 1 during the follow-up period. (D) Dynamic changes of ALT in Cohort 1 during the follow-up period. (E) Dynamic changes of AST in Cohort 1 during the follow-up period. The time points for performing PhIP-seq are marked in red (A-E). (F) Criteria for screening positive peptides. (G) The composition ratio of HBV antigens in the HBV peptide library (left) and positive peptides (right). EF, enrichment factor; FC, functional cure. NR, normalized reads.


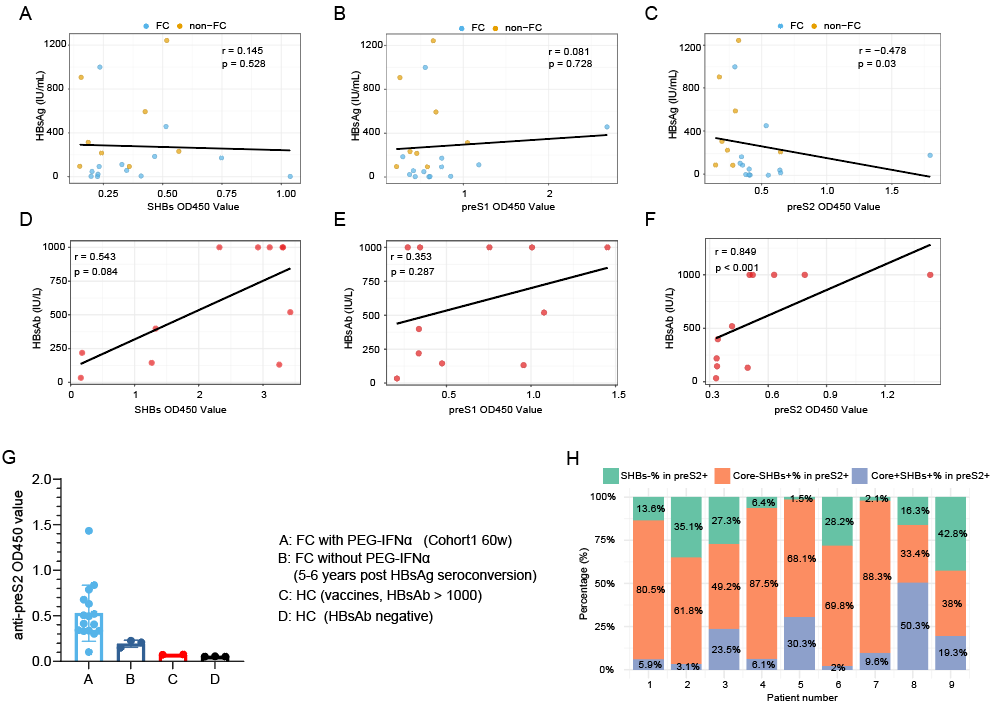
**Figure S2 Correlation of anti-HBV antigen responses or antigen distribution in liver with clinical data.**

(A-C) Correlation between anti-HBV antigen OD450 values and HBsAg levels (above 1 IU/mL) at Baseline. SHBs (A), preS1 (B), and preS2 (C). (D-F) Correlation between anti-HBV antigen OD450 values and HBsAb levels at week 60. SHBs (D), preS1 (E), and preS2 (F). (G) Comparison of anti-preS2 in patients with FC or HC. (H) The composition ratio of SHBs-, SHBs+Core-, and SHBs+Core+ percentage in preS2+ cell. Statistical analysis was performed using Spearman's r test (A-F). FC, functional cure. HC, healthy controls.


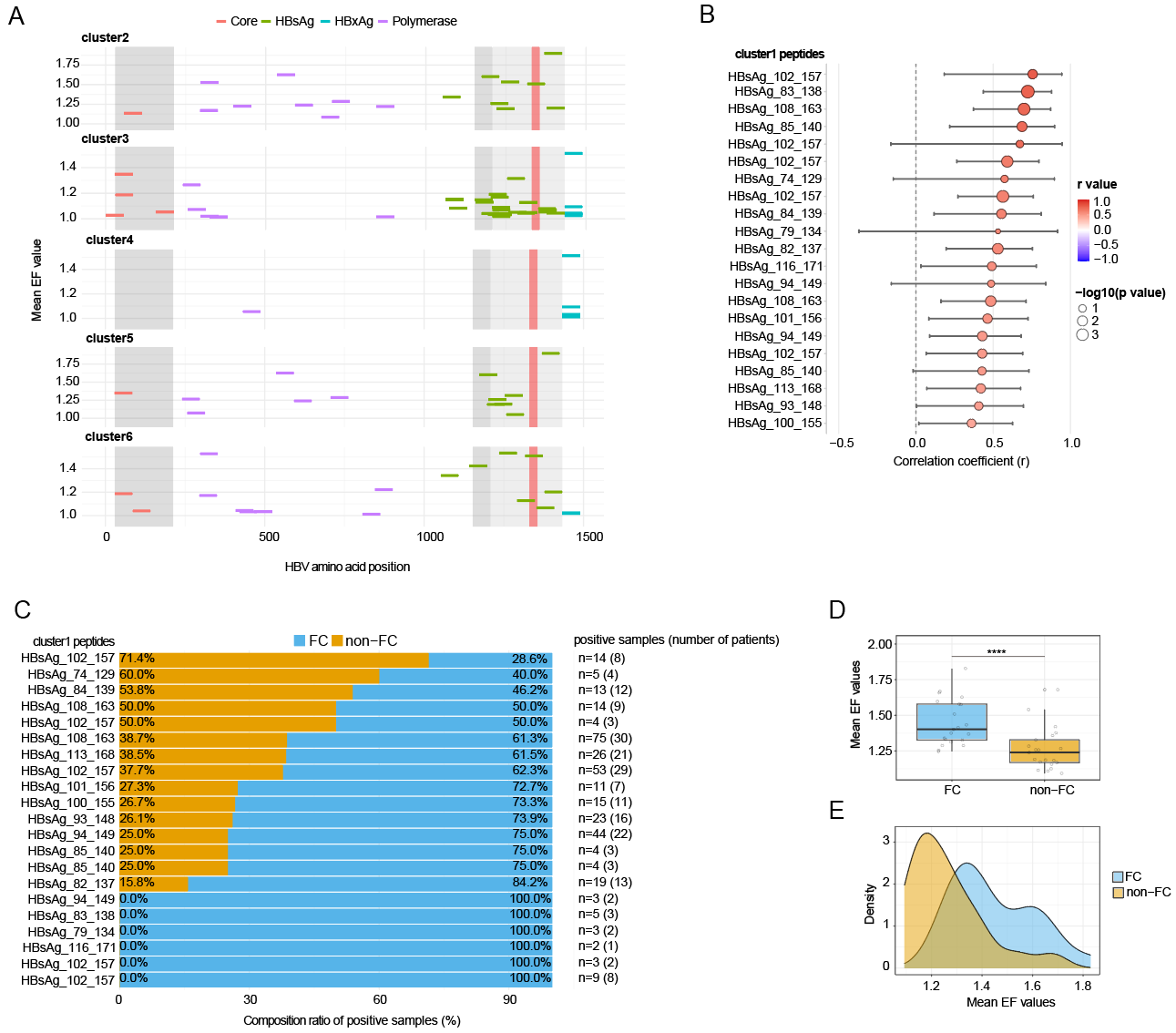


**Figure S3 Distribution, correlation and differences in anti-HBV peptides responses.**

(A) HBV antigen matching characteristics of peptides included in the cluster2–cluster6. (B) Forest plot of correlation analysis between the 21 peptides in cluster1 and the HBsAb level. (C) The composition ratio of positive-reacting plasma samples (raw EF values ＞ 1.5) for 21 peptides in cluster1 in the FC and non-FC group. The numbers in brackets indicate the number of patients with a positive antibody reaction (one patient may have positive-reacting plasma samples at multiple time points). (D) Boxplot of intergroup differences in raw EF values for Cluster 1 peptides. (E) Density of the raw EF values distribution for Cluster 1 peptides in the FC and non-FC groups. Statistical analysis was performed using Spearman's r test (B) and Mann-Whitney U test (D). *p<0.05; **p<0.01; ***p<0.001. EF, enrichment factor; FC, functional cure.


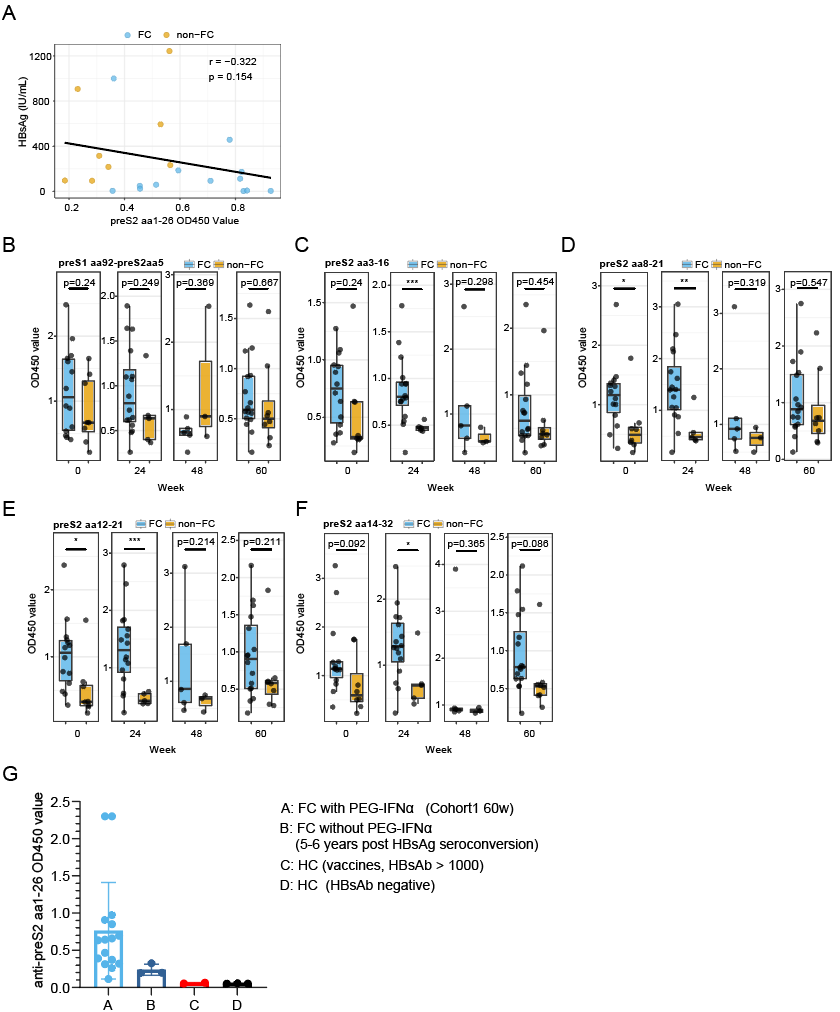


**Figure S4 Differences in the antibody responses targeting different HBsAg antigens between FC and non-FC patients.**

(A) Correlation between anti-preS2 aa1-26 OD450 values and HBsAg levels (above 1 IU/mL) at Baseline. (B-F) The OD values of antibody response at four follow-up time points among FC and non-FC group targeting the preS1 aa92–preS2 aa5, preS2 aa3–16 (B), preS2 aa 8–21 (C), preS2 aa12–21 (D) and preS2 aa14–32 (E). (G) Comparison of anti-preS2 aa1-26 in patients with FC or HC. Statistical analysis was performed using Spearman's r test (A) and t-test (B-F). *p<0.05; **p<0.01; ***p<0.001. FC, functional cure. HC, healthy controls.


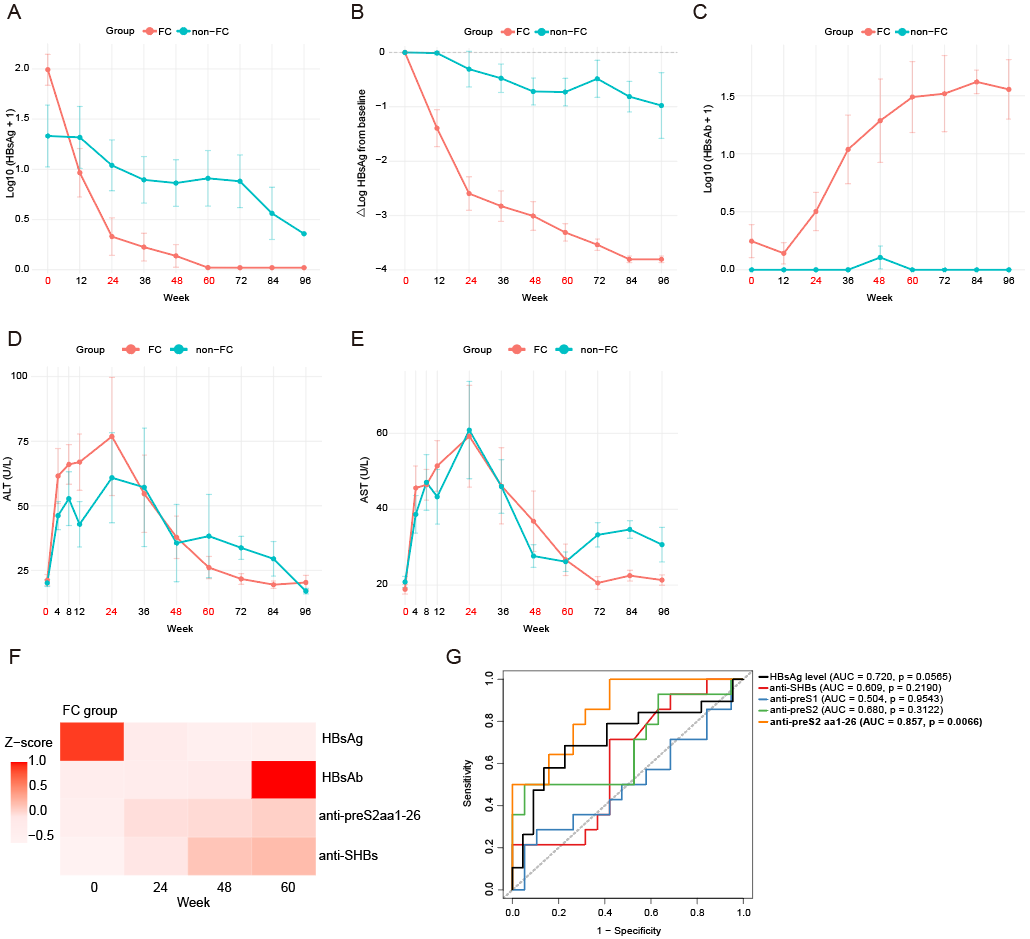


**Figure S5 The dynamic changes of clinical data and antibody responses**

(A) Dynamic changes in HBsAg level of Cohort 2 during the follow-up period. (B) The reduction characteristic of the HBsAg level of Cohort 2 during the follow-up period. (C) Dynamic changes of HBsAb level in Cohort 2 during the follow-up period. (D) Dynamic changes of ALT level in Cohort 2 during the follow-up period. (E) Dynamic changes of AST level in Cohort 2 during the follow-up period. The time points for performing ELISA are indicated in red (A–E). (F) Heatmap of mean Z-score values for HBsAg, HBsAb, anti-SHBs, and preS2 aa1–26 in FC group patients. (G) ROC curves assess the prediction of functional cure in the baseline of PEG-IFN treatment. FC group (n = 19), non-FC group (n = 14). FC, functional cure.


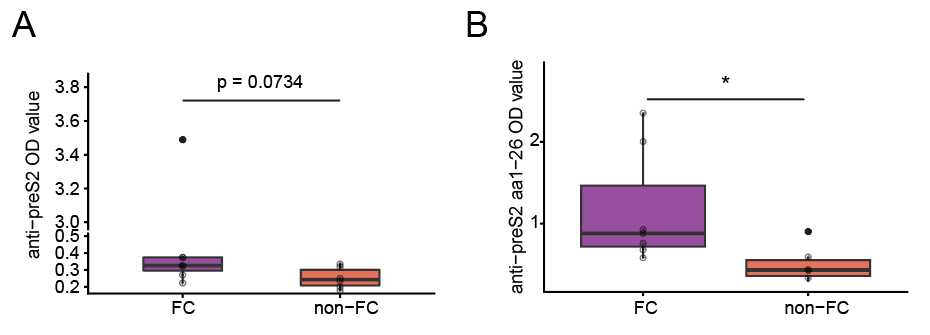


**Figure S6 Differences in antibody responses between FC and non-FC group in the Cohort 3.**

(A-B) The OD value of specific antibody responses against preS2 (A) and preS2 aa 1–26 (B) in different groups. Statistical analysis was performed using Mann-Whitney U test. *p<0.05. FC, functional cure.


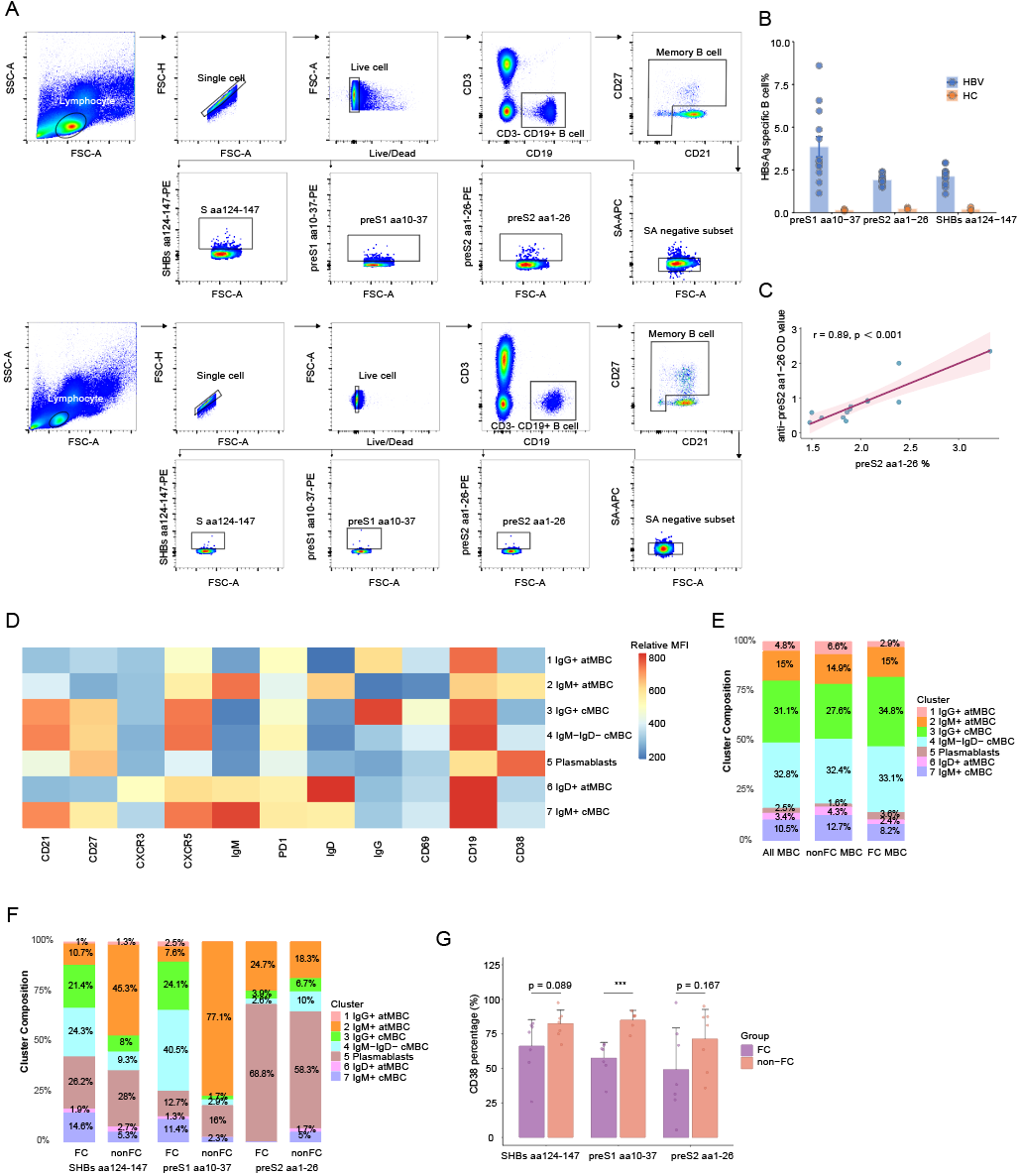


**Figure S7 Gating strategy and phenotypic characteristics of HBV-specific B cells in flow cytometry.**

PBMCs were stained with Live/Dead, anti-CD3, anti-CD19, anti-CD21, anti-CD27, streptavidin-APC, preS1 aa10–37-PE, preS2 aa1–26-PE and SHB aa124–147-PE. (A) Gating strategy for identify HBV epitope-specific B cells in MBCs from HBV infected patients (top) and HC (bottom). (B) The proportions of the three HBsAg-specific B cells in the HBV and HC groups. (C) Correlation analysis between preS2 aa1–26 OD value and preS2 aa1–26 specific B-cell frequencies in Cohort 3. (D) The normalised expression levels of 11 biomarkers used in dimensionality reduction for MBCs across 7 clusters. (E) The composition ratio of 7 MBC clusters and the ratio of MBCs in the FC and non-FC groups. (F) Composition ratio of 7 MBC clusters within the three HBV-specific B cells in the FC and non-FC groups. (G) The proportions of CD38 among the 3 HBsAg-specific B cells in the FC and non-FC groups. Statistical analysis was performed using Pearson's r test (B) and t-test (G). atMBC, atypical memory B cell; cMBC, classical memory B cell; FC, functional cure; HC, healthy controls; MBC, memory B cells; MFI, mean fluorescence intensity.

Table S1 Baseline characteristics of Cohort 1 (n=32)

|  | FC n=18 | Non-FC n=14 | P Value |
| --- | --- | --- | --- |
| Male/female, n | 14/4 | 13/1 | 0.2438 |
| Age (years), median (quartiles) | 38 (31, 46) | 39.5 (37, 46) | 0.6740 |
| Route of transmission, maternal/others, n | 3/15 | 4/10 | 0.419 |
| qHBsAg (IU/mL), median (quartiles) | 53.26 (4.21, 172) | 353.35 (232.2, 798.3) | 0.0085 |
| HBeAg positive/negative | 5/13 | 3/11 | 0.7321 |
| HBeAb positive/negative | 11/7 | 8/6 | 0.8206 |
| ALT (U/L), median (quartiles) | 19.5 (12, 26) | 26 (22, 34) | 0.2092 |
| AST (U/L), median (quartiles) | 20.5 (18, 23) | 24.5 (21.5, 32) | 0.0028 |
| FIB-4, median (quartiles) | 0.715 (0.606, 1.11) | 0.816 (0.748, 1.19) | 0.249 |

Table S2 Baseline clinical characteristics of Cohort 2 (n=19)

|  | FC n=12 | Non-FC n=7 | P Value |
| --- | --- | --- | --- |
| Male/female, n | 8/4 | 6/1 | 0.3631 |
| Age (years), median (quartiles) | 35.5 (32, 43) | 47 (43.25, 48) | 0.0026 |
| qHBsAg (IU/mL), median (quartiles) | 116.4 (58.275, 225) | 32.3 (2.845, 164) | 0.1032 |
| HBeAg positive/negative | 0/12 | 0/7 | - |
| HBeAb positive/negative | 12/0 | 7/0 | - |
| ALT (U/L), median (quartiles) | 22.5 (13, 27.5) | 20.5 (18, 22.25) | 0.7466 |
| AST (U/L), median (quartiles) | 19.5 (15, 20.75) | 21 (20, 24.25) | 0.3369 |

Table S3 Clinical characteristics of FC and non-FC patients in Cohort 3 following PEG-IFNα treatment

|  | Sex  (male/female) | Age (years) | HBsAg (IU/mL) | HBsAb (IU/L) | HBeAg (COI) | HBeAb (COI) | HBcAb (COI) | HBV DNA  (IU/mL) # | ALT (U/L) | AST (U/L) | Sampling time |
| --- | --- | --- | --- | --- | --- | --- | --- | --- | --- | --- | --- |
| FC |  |  |  |  |  |  |  |  |  |  |  |
| 1 | male | 37 | <0.05 | 55.6 | 0.09 | 0.089 | 0.008 | <40 | 42 | 62 | EOT* |
| 2 | male | 40 | <0.05 | <2 | 0.68 | 1.4 | 0.017 | <40 | 20 | 19 | EOT |
| 3 | male | 50 | <0.05 | 388 | 0.08 | 0.427 | 0.009 | <40 | 63 | 57 | EOT |
| 4 | male | 57 | <0.05 | 112 | 0.11 | 1.48 | 0.009 | <40 | 22 | 29 | EOT |
| 5 | female | 49 | <0.05 | 266 | 0.07 | 0.25 | 0.009 | <40 | 11 | 22 | EOT |
| 6 | male | 39 | <0.05 | >1000 | 0.42 | 1.44 | 0.059 | <40 | 17 | 20 | EOT |
| 7 | female | 37 | <0.05 | 62.9 | 0.09 | 1.45 | 0.01 | <40 | 7 | 22 | EOT |
| Non-FC |  |  |  |  |  |  |  |  |  |  |  |
| 1 | male | 35 | 340.6 | <2 | 0.13 | 0.003 | 0.013 | <40 | 37 | 23 | 12 weeks  before EOT |
| 2 | female | 57 | 6.22 | <2 | 0.14 | 0.302 | 0.009 | <40 | 100 | 89 | 12 weeks after EOT |
| 3 | male | 48 | 15.84 | <2 | 0.1 | 1.48 | 0.01 | <40 | 45 | 49 | EOT |
| 4 | male | 34 | 49 | 15.7 | 0.58 | 1.18 | 0.007 | <40 | 26 | 28 | EOT |
| 5 | male | 45 | 0.071 | <2 | 0.29 | 0.003 | 0.01 | <40 | 72 | 65 | EOT |
| 6 | female | 34 | 910 | <2 | 1.29 | 0.941 | 0.009 | <40 | 27 | 34 | EOT |
| 7 | female | 45 | 10.4 | <2 | 0.08 | 0.002 | 0.007 | <40 | 26 | 36 | EOT |
| # Detection Limit of HBV DNA is 40 IU/mL. EOT, end of PEG-IFNα treatment. | | | | | | | | | | | |

Table S4 Clinical characteristics of participants received liver biopsies

|  | Sex (male/female) | Age (years) | HBsAg (IU/mL) | HBsAb (IU/L) | HBeAg (COI) | HBeAb (COI) | HBcAb (COI) | HBV DNA(IU/mL) | ALT (U/L) | AST (U/L) |
| --- | --- | --- | --- | --- | --- | --- | --- | --- | --- | --- |
| CHB |  |  |  |  |  |  |  |  |  |  |
| 1 | male | 21 | 1871 | <2 | 97.7 | 1.66 | 0.007 | 11700000 | 117 | 59 |
| 2 | male | 21 | 3386 | 20.43 | 1160 | 5.77 | 0.009 | 3150000 | 20 | 32 |
| 3 | male | 23 | 101520 | <2 | 1233 | / | 0.007 | 100000000 | 63 | 27 |
| 4 | female | 37 | 4911 | <2 | 79.33 | 0.909 | 0.009 | 6130 | 20 | 26 |
| 5 | female | 40 | 3489 | <2 | 1354 | / | 0.006 | 13900000 | 239 | 163 |
| 6 | male | 40 | 5708 | <2 | 95.37 | 1.34 | 0.009 | 2580000 | 709 | 178 |
| 7 | female | 50 | 21478 | 63.6 | 1507 | 5.19 | 0.007 | 113 | 55 | 78 |
| 8 | male | 54 | 7172 | <2 | 1008 | 5 | 0.011 | 27000000 | 586 | 274 |
| 9 | male | 59 | 808 | <2 | 749 | 3.12 | 0.007 | 12000000 | 288 | 146 |

Table S5 Clinical characteristics of participants without PEG-IFNα therapy (FC without PEG-IFNα) and healthy controls (HC)

|  | Sex (male/female) | Age (years) | HBsAg (IU/mL) | HBsAb (IU/L) | HBeAg (COI) | HBeAb (COI) | HBcAb (COI) | HBV DNA (IU/mL) | ALT (U/L) | AST (U/L) |
| --- | --- | --- | --- | --- | --- | --- | --- | --- | --- | --- |
| FC without PEG-IFNα | |  |  |  |  |  |  |  |  |  |
| 1 | male | 60 | <0.05 | 122 | 0.11 | 1.12 | 0.008 | <40 | 15 | 20 |
| 2 | male | 63 | <0.05 | 11.6 | 0.16 | 1.32 | 0.008 | <40 | 22 | 22 |
| 3 | male | 52 | <0.05 | 104 | 0.13 | 0.041 | 0.008 | <40 | 33 | 30 |
| HC | |  |  |  |  |  |  |  |  |  |
| 1 | female | 47 | - | >1000 | - | - | - | - | - | - |
| 2 | female | 33 | - | >1000 | - | - | - | - | - | - |
| 3 | male | 24 | - | <2 | - | - | - | - | - | - |
| 4 | male | 29 | - | <2 | - | - | - | - | - | - |
| 5 | female | 36 | - | <2 | - | - | - | - | - | - |

Table S6 HBV peptide sequences used in the PhIP-seq library

Data are shown in supplementary materials: HBV Virus Lib peptides.xlsx

Table S7 List of antibodies used for flow cytometry experiments

| Specificity | Fluorochrome | Clone | Purpose | Manufacturer | Cat # |
| --- | --- | --- | --- | --- | --- |
| CD3 | BV605 | SK7 | Negative gate | Biolegend | 344836 |
| CD19 | BV737 | SJ25C1 | Total B cell | BD | 612756 |
| CD21 | PerCP-eFluor 710 | HB5 | Memory B cell | Invitrogen | 46-0219-42 |
| CD27 | FITC | O323 | Memory B cell | Invitrogen | 11-0279-42 |
| CD38 | PE-Cy7 | HB7 | Plasmablast | Biolegend | 356608 |
| IgM | BV650 | G20-127 | B cell class-switch | BD | 740595 |
| IgG | BUV395 | G18-145 | B cell class-switch | BD | 564229 |
| IgD | BV785 | IA6-2 | B cell class-switch | Biolegend | 348242 |
| CXCR3 | BV421 | G025H7 | Chemokine receptors | Biolegend | 353716 |
| CXCR5 | BV510 | RF8B2 | Chemokine receptors | BD | 563105 |
| CD69 | BUV496 | FN50 | Activation | BD | 750214 |
| PD-1 | BV711 | NAT105 | Exhaustion | Biolegend | 367428 |
| Fixable Viability Stain | AF700 | / | Live/dead | BD | 564997 |
| / | PE | / | HBV specific B cell tag | Agilent | PJRS25-1 |
| / | APC | / | Negative gate | Agilent | PJ27S-1 |

Table S8 Peptide sequence of cluster1

| No. | Position | Sequence |
| --- | --- | --- |
| 1 | HBsAg_74_129 | LTTVPATPPPASTNRQSGRKPTPLSPPLRDTHPQAMQWNSNTFHQTLQDPRVRALY |
| 2 | HBsAg_79_134 | AAPPPASTNRQSGRQPTPLSPPLRDTHPQAMQWNSTTFHQTLQDPRVRALYFPAGG |
| 3 | HBsAg_82_137 | SSCLHQSAVRKAAYSLISTSRDTHPQAMQWNSTTFHQTLQDPRVRALYFPAGGSSS |
| 4 | HBsAg_83_138 | PASTIRQSGRQPTPISPPLRDSHPQAMRWNSTTFHQALQDPRIRGLYFPAGGSSSG |
| 5 | HBsAg_84_139 | ASTNRQTGRQPTPLSPPLRDTHPQAMQWNSTTFHQTLQDPRVRALYFPAGGSVQEQ |
| 6 | HBsAg_85_140 | STNRQSGRQPTPLSPPLRDTHPQAMQWNSTTFHQALQDPRVRGLYFPAGGSSSGTL |
| 7 | HBsAg_85_140 | STNRQSGRQPTPLSPPLRNTHPQAMQWNSTTFHQTLQDPRVRGLYFPAGGSSSGTV |
| 8 | HBsAg_93_148 | QPTPISPPLRDTHPQAMQWNSTTFHQALQDPRVRALYFPAGGSSSGTVSPAQNTVS |
| 9 | HBsAg_94_149 | LTTVPAAPPPASTNRQSGNSTTFHQTLQDPRVRALYFPAGGSSSGTVSPAQNTASA |
| 10 | HBsAg_94_149 | PTPISPPLRDTHPQAMQWNSTTFHQTLQDPRVRALYFPAGGSSSGTVNPAQNTASS |
| 11 | HBsAg_100_155 | STNRQSGQAMQWNSTTFHQTLQDPRVRGLYFPAGGSSSGTVNPVPTTVSHISSIFS |
| 12 | HBsAg_101_156 | LRDSHPQAMQWNSTAFHQALQDPRVRGLYFPAGGSSSGTLNPVPTIASHISSISSR |
| 13 | HBsAg_102_157 | RDTHPQAMQWNSTTFHQTLQDPRVRALYLPAGGSSSGTVSPAQNTVSAISSILSTT |
| 14 | HBsAg_102_157 | RDTHPQAMQWNSTTFHQTLQDPRVRALYFPAGGSSSGTVSPAQNTVSAISSTLSKT |
| 15 | HBsAg_102_157 | RDTHPQAMQWNSNTFHQTLQDPRVRALYFPAGGSSSGTVSPAQNTASAISSTFSKT |
| 16 | HBsAg_102_157 | RDSHPQAMQWNSTTFHQALLDPRVRGLYFPAGGSSSGTANPVPTTASPISSIFSRT |
| 17 | HBsAg_102_157 | RDSHPQAMQWNSSTFHQALLDPRVRGLYFPAGGSSSGTVNPVPTTASPISSIFSRT |
| 18 | HBsAg_108_163 | SMQWNSTTFHQTLQDPRVRALYFPAGGSSSGTVNPVQTTASSILSISSTTGDPVPN |
| 19 | HBsAg_108_163 | SMQWNSTTFHQALLDPRVRGLYFPAGGSSSGTVNPVQTTASSISSTLSKTGDPVPN |
| 20 | HBsAg_113_168 | STTFHQTLQDPRVRALYFPAGGSSSGTVNPVQNTASSISSILSTTGDPVPNMENIA |
| 21 | HBsAg_116_171 | FHQTLQDPRVRALYFPAGGSSSGTVSPAQNTVSAISSILSKTGTLYRTWRTSHQDS |

Table S9 Matching characteristics of the cluster1 peptides in Uniprot database

| No. | Entry | Organism | HBV  genotype | Identity (%) |
| --- | --- | --- | --- | --- |
| 1 | Q9PWW3 | Hepatitis B virus genotype B2 (isolate Vietnam/16091/1992) (HBV-B) | B | 94.6% |
| 2 | **P17398** | Hepatitis B virus genotype B1 subtype adw (isolate Japan/pJDW233/1988) (HBV-B) | B | 100% |
| 3 | P17397 | Hepatitis B virus genotype B2 (isolate Indonesia/pIDW420/1988) (HBV-B) | B | 100% |
| 4 | Q02317 | Hepatitis B virus genotype A1 subtype adw (isolate Philippines/pFDW294/1988) (HBV-A) | A | 92.9% |
| 5 | **P17398** | Hepatitis B virus genotype B1 subtype adw (isolate Japan/pJDW233/1988) (HBV-B) | B | 98.1% |
| 6 | P12911 | Hepatitis B virus (isolate United Kingdom/LSH/1988) (HBVcpz) | - | 100% |
| 7 | P03138 | Hepatitis B virus genotype D subtype ayw (isolate France/Tiollais/1979) (HBV-D) | D | 100% |
| 8 | **P17398** | Hepatitis B virus genotype B1 subtype adw (isolate Japan/pJDW233/1988) (HBV-B) | B | 96.4% |
| 9 | **P17398** | Hepatitis B virus genotype B1 subtype adw (isolate Japan/pJDW233/1988) (HBV-B) | B | 71.1% |
| 10 | P17397 | Hepatitis B virus genotype B2 (isolate Indonesia/pIDW420/1988) (HBV-B) | B | 96.4% |
| 11 | Q80IU3 | Hepatitis B virus genotype E (isolate Cote d'Ivoire/ABI-212/2003) (HBV-E) | E | 76.1% |
| 12 | Q02317 | Hepatitis B virus genotype A1 subtype adw (isolate Philippines/pFDW294/1988) (HBV-A) | A | 100% |
| 13 | P03140 | Hepatitis B virus genotype C subtype ad (isolate Japan/S-179/1988) (HBV-C) | C | 98.2% |
| 14 | Q9E6S4 | Hepatitis B virus genotype C (isolate Vietnam/3270/2000) (HBV-C) | C | 100% |
| 15 | Q9QAB7 | Hepatitis B virus genotype B2 (isolate Vietnam/9873/1997) (HBV-B) | B | 94.6% |
| 16 | Q9QAB7 | Hepatitis B virus genotype B2 (isolate Vietnam/9873/1997) (HBV-B) | B | 100% |
| 17 | Q8JXB9 | Hepatitis B virus genotype B1 (isolate Japan/Ry30/2002) (HBV-B) | B | 100% |
| 18 | Q76R62-2 | Hepatitis B virus genotype C subtype ayr (isolate Human/Japan/Okamoto/-) (HBV-C) | C | 89.1% |
| 19 | P17397 | Hepatitis B virus genotype B2 (isolate Indonesia/pIDW420/1988) (HBV-B) | B | 92.9% |
| 20 | P17397 | Hepatitis B virus genotype B2 (isolate Indonesia/pIDW420/1988) (HBV-B) | B | 100% |
| 21 | **P17398** | Hepatitis B virus genotype B1 subtype adw (isolate Japan/pJDW233/1988) (HBV-B) | B | 100% |

Table S10 Synthesised peptides used for ELISA validation experiment

| No. | Length | Sequence | Reference (PMID) |
| --- | --- | --- | --- |
| 1 | 22 | RQPTPLSPPLRDTHPQAMQWNS | 1998 J Virol  (9621015) |
| 2 | 26 | MQWNSTTFHQTLQDPRVRALYFPAGG | 1986 Vaccine  (2421497) |
| 3 | 19 | DPRVRALYFPAGGSSSGTV | 1986 Proc Natl Acad Sci USA (3466181) |
| 4 | 14 | WNSTTFHQTLQDPR | 2000 J Gen Virol (10644835) |
| 5 | 10 | LQDPRVRALY | 1992 J Biotechnol  (1369362) |
| 6 | 14 | FHQTLQDPRVRALY | Current study |
